# Physiological and Metabolic Effects of Yellow Mangosteen (*Garcinia dulcis*) Rind in Rats with Diet-Induced Metabolic Syndrome

**DOI:** 10.3390/ijms21010272

**Published:** 2019-12-31

**Authors:** Oliver D. John, Peter Mouatt, Marwan E. Majzoub, Torsten Thomas, Sunil K. Panchal, Lindsay Brown

**Affiliations:** 1 Functional Foods Research Group, University of Southern Queensland, Toowoomba, QLD 4350, Australia; oliverdjohn@outlook.com (O.D.J.); skpanchal2001@gmail.com (S.K.P.); 2 School of Health and Wellbeing, University of Southern Queensland, Toowoomba, QLD 4350, Australia; 3 Southern Cross Plant Science, Southern Cross University, Lismore, NSW 2480, Australia; Peter.Mouatt@scu.edu.au; 4 Centre for Marine Science and Innovation & School of Biological, Earth and Environmental Sciences, University of New South Wales, Sydney, NSW 2052, Australia; m.majzoub@unsw.edu.au (M.E.M.); t.thomas@unsw.edu.au (T.T.)

**Keywords:** metabolic syndrome, *Garcinia dulcis*, citric acid, garcinol, morelloflavone, microbiota

## Abstract

Metabolic syndrome is a cluster of disorders that increase the risk of cardiovascular disease and diabetes. This study has investigated the responses to rind of yellow mangosteen (*Garcinia dulcis*), usually discarded as waste, in a rat model of human metabolic syndrome. The rind contains higher concentrations of phytochemicals (such as garcinol, morelloflavone and citric acid) than the pulp. Male Wistar rats aged 8–9 weeks were fed either corn starch diet or high-carbohydrate, high-fat diet for 16 weeks, which were supplemented with 5% freeze-dried *G. dulcis* fruit rind powder during the last 8 weeks. We characterised metabolic, cardiovascular, liver and gut microbiota parameters. High-carbohydrate, high-fat diet-fed rats developed abdominal obesity, hypertension, increased left ventricular diastolic stiffness, decreased glucose tolerance, fatty liver and reduced *Bacteroidia* with increased *Clostridia* in the colonic microbiota. *G. dulcis* fruit rind powder attenuated these changes, improved cardiovascular and liver structure and function, and attenuated changes in colonic microbiota. *G. dulcis* fruit rind powder may be effective in metabolic syndrome by appetite suppression, inhibition of inflammatory processes and increased fat metabolism, possibly related to changes in the colonic microbiota. Hence, we propose the use of *G. dulcis* fruit rind as a functional food to ameliorate symptoms of metabolic syndrome.

## 1. Introduction

The genus *Garcinia* includes more than 250 species of shrubs and trees of the Clusiaceae or Guttiferae family that grow in lowland tropical forests. Species include *G. mangostana* (purple mangosteen), *G. cambogia* (Malabar tamarind) and *G. humilis* (achacha or Bolivian mangosteen) [1]. The yellow mangosteen or *G. dulcis* (Roxb.) Kurz is native to Borneo, Java, the Philippines, Malaya and southern Thailand and cultivated throughout Southeast Asia and the American tropical regions [2]. In Australia, *G. dulcis* is usually restricted to north Queensland, where it grows in rainforests from the Melville range, Cape York Peninsula, to the Torres Strait islands [3] but is also grown around South East Queensland [4]. The young fruit is green but turns yellow when ripe. The ripe juicy pulp tastes sour to sweet [5]. The crushed fruit is used as an expectorant to clear sputum, alleviate cough, treat scurvy and as a mild laxative [2,5,6]. An extract of *G. dulcis* fruit showed hepatoprotective and anti-proliferative properties [7,8].

The major bioactive compounds in the fruits of *G. dulcis* were morelloflavone and garcinol [9]. Morelloflavone has hypocholesterolaemic and anti-atherogenic properties [10,11]. Garcinol showed anti-inflammatory, anti-oxidant, anti-cancer, anti-diabetic and cardioprotective properties and protection against allergy and neurodegenerative diseases [12]. In addition, morelloflavone, garcinol and xanthone derivatives in *G. dulcis* have anti-inflammatory, anti-oxidant, anti-viral, anti-cancer and hypocholesterolaemic properties [2,9].

Metabolic syndrome is a collection of symptoms such as abdominal obesity, hypertension, dyslipidaemia, insulin resistance and a pro-thrombotic state [13,14]. Abdominal obesity was measured by increased waist circumference; dyslipidaemia was measured by increased plasma triglycerides and reduced HDL-cholesterol concentrations, and insulin resistance was measured by high fasting plasma glucose and insulin concentrations. Further, changes in the composition of the gut microbiota have been associated with the pathogenesis of metabolic syndrome [15]. Increased consumption of fruit and vegetables could prevent chronic diseases such as cardiovascular disease and prevent body weight gain [16]. Additionally, plant-based foods reduced metabolic syndrome risk [17].

In this study, we evaluated the reversal by *G. dulcis* rind of the metabolic, cardiovascular, liver and gut microbiota changes produced by intervention with food containing high simple sugars and saturated and *trans* fats in rats. This model mimics metabolic syndrome in humans [18]. The rind of *Garcinia* fruits, usually regarded as a waste product, was chosen because of its higher concentrations of potentially bioactive phytochemicals compared to the pulp [19,20]. Parameters that were analysed included metabolic parameters such as body weight, fat deposition, lipid profiles, adipocyte histology, glucose tolerance and the gut microbiota, as well as cardiovascular and liver parameters such as blood pressure, left ventricular diastolic stiffness, plasma liver enzymes and organ structure. We hypothesised that *G. dulcis* rind will reverse the symptoms in diet-induced rat model of metabolic syndrome consistent with the reported bioactivity of garcinol, morelloflavone and citric acid that are present in the rind.

## 2. Results

### 2.1. Weight and Phytochemical Analysis of *G. dulcis* Rind, Pulp and Seed

**Figure 1:**
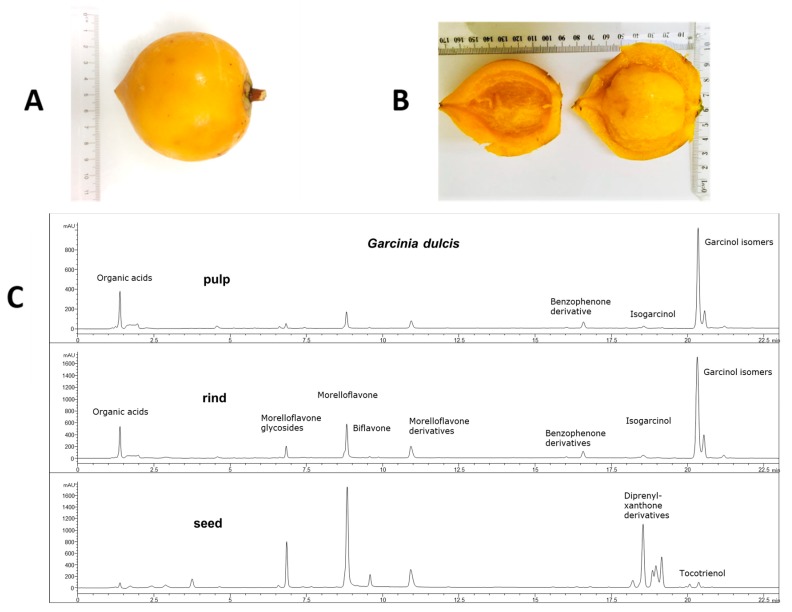
(**A**) Whole *Garcinia dulcis* fruit, (**B**) *Garcinia dulcis* rind and pulp and (**C**) high-performance liquid chromatography profile at 210 nm of *Garcinia dulcis* pulp, rind and seed.

The ripe fruit was about 8 cm in diameter with 1 cm thick rind and weighed 161 ± 7 g (*n* = 6; Figure 1A,B). Wet weights of the rind, pulp and seed were 64 ± 3 g, 62 ± 4 g and 35 ± 1 g, respectively. The total moisture contents of the rind, pulp and seed were 83%, 82% and 49%, respectively. The high-performance liquid chromatography (HPLC) chromatograms for pulp, rind and seed are given in Figure 1C. The rind contained benzophenones (7.86% *w*/*w*) including garcinol (6.35% *w*/*w*), as well as citric acid (24% *w*/*w*) and morelloflavone (0.55% *w*/*w*). The pulp contained benzophenones (3.54% *w*/*w*) including garcinol (2.87% *w*/*w*), as well as citric acid (19% *w*/*w*) and morelloflavone (0.11% *w*/*w*). The seeds contained morelloflavone (2.9%) and diprenyl-xanthone derivatives, but citric acid and benzophenones were not detected.

### 2.2. Metabolic Parameters

The consumption of food in high-carbohydrate, high-fat diet-fed rats (H) was lower than in corn starch diet-fed rats (C) (Table 1). This translates to lower intakes of garcinol, morelloflavone and citric acid in high-carbohydrate, high-fat diet-fed rats treated with *G. dulcis* (HGD) compared to corn starch diet-fed rats treated with *G. dulcis* (CGD) (Table 1). *G. dulcis* treatment reduced food intake in CGD and HGD rats, but water intake was unchanged (Table 1). The energy intakes in H rats were higher than in C rats due to the higher energy density of high-carbohydrate, high-fat diet, but HGD and CGD rats had lower energy intake than H and C rats, respectively, due to decreased food intake (Table 1).

**Figure 2:**
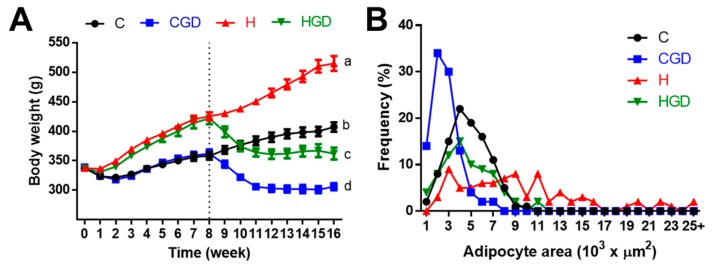
(**A**) Weekly body weight of C, CGD, H and HGD rats; dotted line signifies when treatment started for CGD and HGD rats, (**B**) Distribution of retroperitoneal adipocyte sizes. C, corn starch diet-fed rats; CGD, corn starch diet-fed rats supplemented with 5% *Garcinia dulcis* rind powder; H, high-carbohydrate, high-fat diet-fed rats; HGD, high-carbohydrate, high-fat diet-fed rats supplemented with 5% *Garcinia dulcis* rind powder.

**Figure 3:**
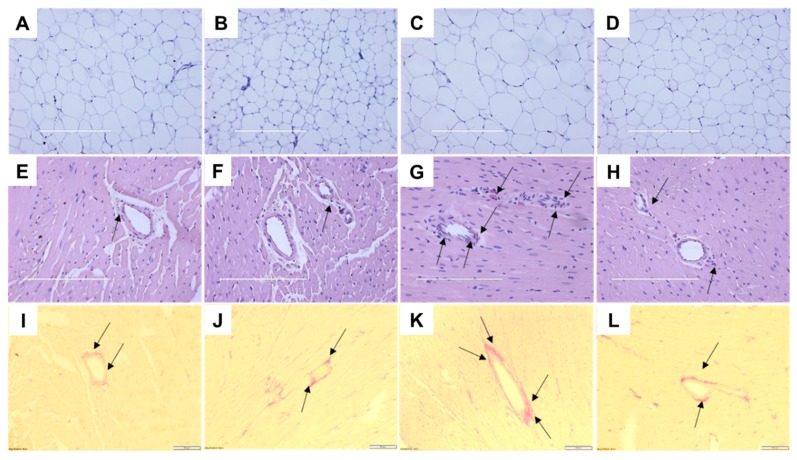
Haematoxylin and eosin staining of adipocytes (**A**–**D**; magnification ×10; scale bar = 400 µm); haematoxylin and eosin staining of left ventricle indicating inflammatory cells as dark spots outside myocytes and in vascular endothelium marked as arrow (**E**–**H**; magnification ×20; scale bar = 200 µm); and picrosirius red staining of left ventricle indicating collagen deposition as red stain marked by arrows (**I**–**L**; magnification ×12.6; scale bar = 100 µm). Corn starch diet-fed rats (**A**,**E**,**I**), corn starch diet-fed rats treated with *Garcinia dulcis* rind powder (**B**,**F**,**J**), high-carbohydrate, high-fat diet-fed rats (**C**,**G**,**K**) and high-carbohydrate, high-fat diet-fed rats treated with *Garcinia dulcis* rind powder (**D**,**H**,**L**).

At 16 weeks, H rats showed increased abdominal fat pads, abdominal circumference and total body fat mass (Table 1). Both HGD and CGD rats showed declining body weights from the beginning of the treatment period (Figure 2A). HGD rats at 16 weeks had lower body weight (Figure 2A), feed efficiency, abdominal circumference, retroperitoneal fat, epididymal fat, omental fat, total abdominal fat, visceral adiposity index, bone mineral content, bone mineral density and body mass index compared to H rats (Table 1). Similarly, CGD rats had lower body weight (Figure 2A), feed efficiency, abdominal circumference, body fat mass, retroperitoneal fat, epididymal fat, omental fat, total abdominal fat and visceral adiposity index compared to C rats (Table 1). Mean adipocyte area was higher in H rats compared to C rats; adipocyte area was lower in both CGD and HGD rats (Table 1; Figure 2B and Figure 3A–D). The adipocyte area distribution showed more adipocytes with larger area in H rats compared to HGD, C and CGD rats (Table 1, Figure 2B).

After 8 weeks, oral glucose response and insulin response indicated insulin resistance in H rats compared to C rats (Table 1). At 16 weeks, HGD showed improvement in both oral glucose response and insulin response while CGD rats showed only reduction in glucose response but not insulin response compared to C rats (Table 1). Plasma catalase activity was increased in H rats compared to C rats and was unchanged by the treatment (Table 1). Faecal lipid content was higher in H and HGD rats compared to C rats and lower in CGD rats (Table 1).

Intervention with *G. dulcis* decreased heat production in both CGD and HGD rats compared to C and H rats, respectively (Table 1). Respiratory exchange ratio values in C and H rats were consistent with the foods and were unchanged by *G. dulcis* intervention (Table 1). Total plasma cholesterol concentrations in CGD rats were lower than C, H and HGD rats. HGD rats had lower concentrations of triglycerides and non-esterified fatty acids than H rats (Table 1).

### 2.3. Cardiovascular Structure and Function

H rats developed an increased systolic blood pressure at weeks 8 and 16 compared to C rats (Table 1). At week 16, HGD rats had lower systolic blood pressure compared to H rats (Table 1). Left ventricular diastolic stiffness was lower in HGD rats than in H rats, which indicated that *G. dulcis* treatment improved heart function (Table 1). Infiltration of inflammatory cells and collagen deposition were lower in the left ventricles of C and CGD rats (Figure 3E,F,I,J). Infiltration of inflammatory cells and perivascular collagen deposition was higher in H rats and lower in HGD rats to be similar to C and CGD rats (Figure 3G,H,K,L).

### 2.4. Liver Structure and Function

**Figure 4:**
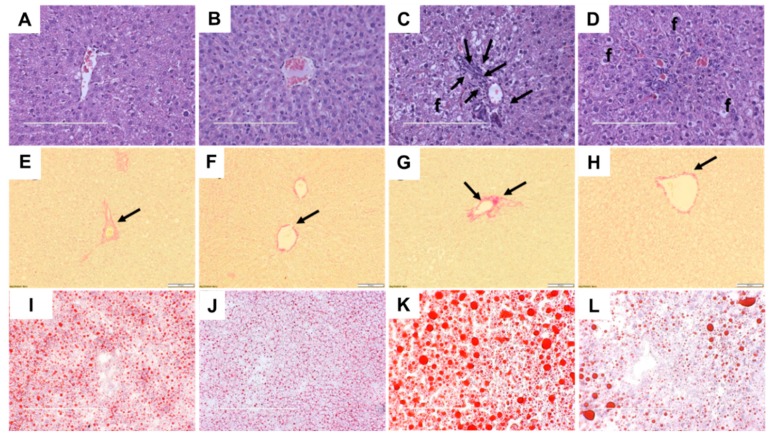
Haematoxylin and eosin staining of liver indicating inflammatory cells as dark spots marked by arrow and fat vacuoles marked as “f”) (**A**–**D**; magnification ×20; scale bar = 200 µm); picrosirius red staining of liver indicating collagen deposition as red stain around blood vessels marked by arrows (**E**–**H**; magnification ×12.6; scale bar = 100 µm); and oil red O stain of liver showing fat droplets in red (**I**–**L**; magnification ×12.6; scale bar = 200 µm). Corn starch diet-fed rats (**A**,**E**,**I**), corn starch diet-fed rats treated with *Garcinia dulcis* rind powder (**B**,**F**,**J**), high-carbohydrate, high-fat diet-fed rats (**C**,**G**,**K**) and high-carbohydrate, high-fat diet-fed rats treated with *Garcinia dulcis* rind powder (**D**,**H**,**L**).

Compared to C rats, H rats showed higher infiltration of inflammatory cells in the liver (Figure 4A,C); infiltration was reduced in HGD rats (Figure 4D). Liver fat deposition was higher in H rats but much reduced in HGD rats (Figure 4C,D). C and CGD rats had minimal liver fat deposition (Figure 4A,B). Picrosirius red staining showed lower collagen deposition in C, CGD and HGD liver, notably in perivascular area (Figure 4E,F,H). Oil red O staining confirmed the reduced fat deposition in C, CGD and HGD liver (Figure 4I,J,L). Liver glycogen content was reduced in CGD and HGD rats compared to C and H rats, respectively (Table 1). Plasma alanine transaminase activities were unchanged by diet or intervention, but aspartate transaminase activities were lower in HGD rats compared to H rats (Table 1).

### 2.5. Gut Structure and Microbiota

**Figure 5:**
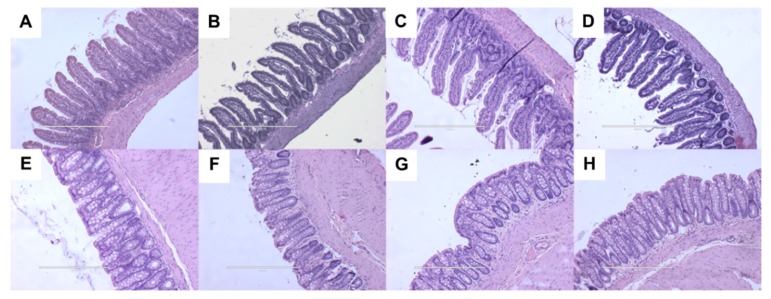
Haematoxylin and eosin staining of ileum (**A**–**D**) and colon (**E**–**H**; magnification ×20; scale bar = 200 µm) in corn starch diet-fed rats (**A**,**E**), corn starch diet-fed rats treated with *Garcinia dulcis* rind powder (**B**,**F**), high-carbohydrate, high-fat diet-fed rats (**C**,**G**) and high-carbohydrate, high-fat diet-fed rats treated with *Garcinia dulcis* rind powder (**D**,**H**).

Gastrointestinal histology showed no differences between the groups, demonstrated by normal crypt depth, villi length, goblet cells and lack of inflammatory cell infiltration (Figure 5).

**Figure 6:**
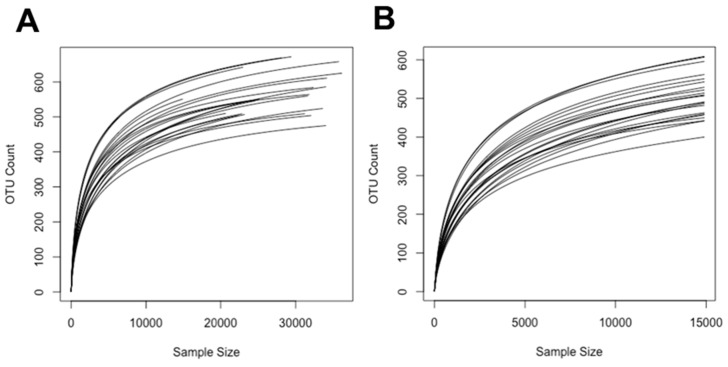
Rarefaction curves (**A**) before and (**B**) after normalisation (14,908 sequences).

We used a 16S rRNA gene-based analysis to assess bacterial communities from rat faecal pellets. Data was obtained for five replicates of control samples (C and H) and six replicates for samples from rats treated with *G. dulcis* (CGD and HGD). After quality filtering, there were a total of 625,711 sequences, and these were clustered into 1114 zero-radius operational taxonomic units (zOTUs). The calculated rarefaction curves based on rarefied and unrarefied data as well as Good’s coverage of 99.68 ± 0.12% revealed that the majority of the bacterial community was recovered by the surveying effort (Figure 6).

#### 2.5.1. Alpha Diversity Measures (Shannon Diversity and Richness) of the Bacterial Communities

**Figure 7:**
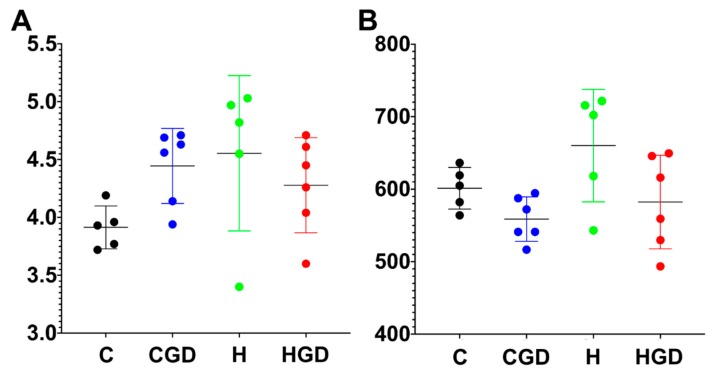
Shannon diversity (**A**) and richness (**B**) of faecal samples. C, corn starch diet-fed rats; CGD, corn starch diet-fed rats treated with *Garcinia dulcis* rind powder; H, high-carbohydrate, high-fat diet-fed rats; HGD, high-carbohydrate, high-fat diet-fed rats treated with *Garcinia dulcis* rind powder.

There was no statistical support for differences in Shannon’s diversity for the four different dietary groups (*p* > 0.05) (Figure 7). There was no statistical support for any pairwise comparison of richness between the dietary groups, except for some marginal support for a higher richness in H rats compared to CGD rats (Figure 7; *p* = 0.0296).

#### 2.5.2. Bacterial Community Structure

**Figure 8:**
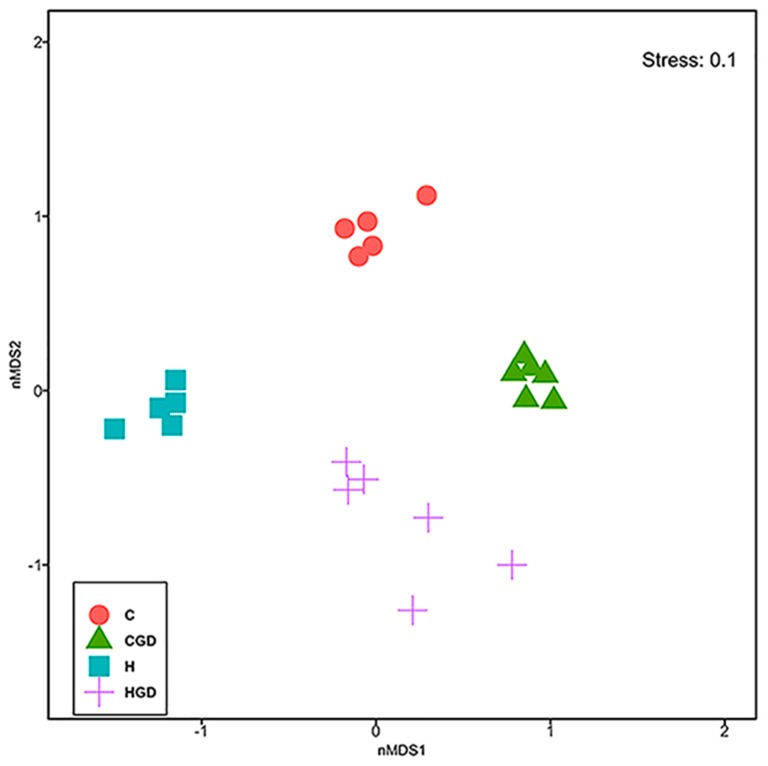
Non-metric, multi-dimensional scaling (nMDS) plot of bacterial community structure of faecal samples from C, CGD, H and HGD rats. C, corn starch diet-fed rats; CGD, corn starch diet-fed rats treated with *Garcinia dulcis* rind powder; H, high-carbohydrate, high-fat diet-fed rats; HGD, high-carbohydrate, high-fat diet-fed rats treated with *Garcinia dulcis* rind powder.

An overall effect of diet (Figure 8, Table 2; Permutational multivariate analysis of variance (PERMANOVA), *p* = 0.0001) and treatment (Figure 8, Table 2; PERMANOVA, *p* = 0.0001) was observed on the overall bacterial community structure based on Bray–Curtis dissimilarity, as well as the interaction of the two factors (diet and treatment) (Figure 8, Table 2; PERMANOVA, *p* = 0.0001).

Analysis of the colonic microbiota showed differences between C and H faecal samples without *G. dulcis* rind powder (*p* = 0.0074) indicating an effect of basal diet on the bacterial community structure. Addition of *G. dulcis* rind powder to either of the diets produced similar changes to the community structure (*p* = 0.0019 to 0.0024 for C and H, respectively) (Figure 8). Bacterial communities in HGD rats were also more variable between replicates compared to H rats (Figure 8, Table 2; PERMDISP; *p* = 0.0338).

#### 2.5.3. Firmicutes and Bacteroidetes Ratio as an Indicator for Obesity

**Figure 9:**
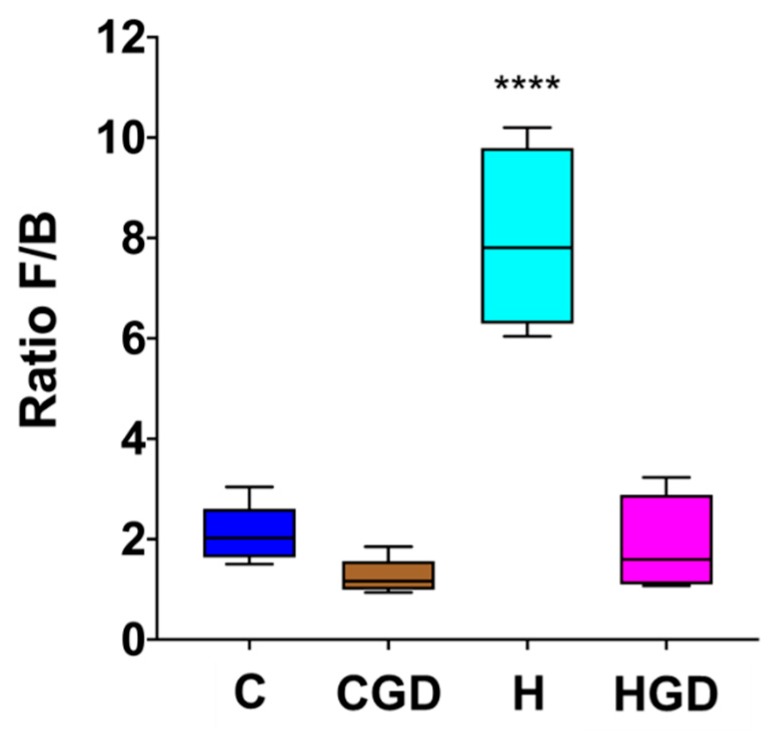
Effect of supplementation of diet (C or H) with *Garcinia dulcis* rind powder on the ratio of Firmicutes and Bacteroidetes (F/B) abundances in rat faecal samples. C, corn starch diet-fed rats; CGD, corn starch diet-fed rats treated with *Garcinia dulcis* rind powder; H, high-carbohydrate, high-fat diet-fed rats; HGD, high-carbohydrate, high-fat diet-fed rats treated with *Garcinia dulcis* rind powder. A one-way ANOVA was performed, significant differences were observed between H samples and the other treatments (C, CGD and HGD; *p* < 0.0001, ****).

Differences in the bacterial community structure were also assessed by calculating the ratio of Firmicutes to Bacteroidetes (F/B ratio). This ratio has previously been found to positively correlate with obesity in humans and other mammals. The data shows that diet (C or H) supplemented with *G. dulcis* resulted in a reduction in F/B ratio, and this difference was more prevalent for H diet (*p* < 0.0001) (Figure 9).

#### 2.5.4. Taxonomic Structure of the Bacterial Communities

**Figure 10:**
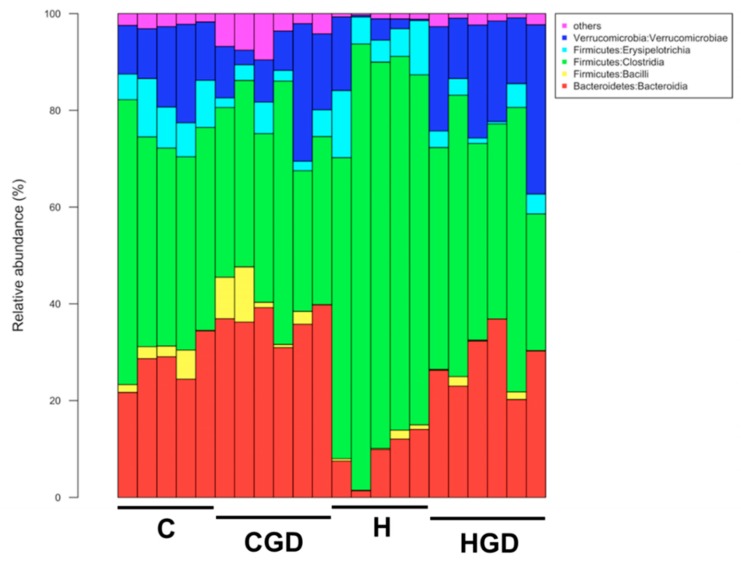
Taxonomic profiles of bacterial communities shown at the class level. C, corn starch diet-fed rats; CGD, corn starch diet-fed rats treated with *Garcinia dulcis* rind powder; H, high-carbohydrate, high-fat diet-fed rats; HGD, high-carbohydrate, high-fat diet-fed rats treated with *Garcinia dulcis* rind powder.

The most abundant bacterial classes found in the faecal samples for different treatment groups belonged to the classes Bacteroidia, Bacilli, Clostridia, Erysipelotrichia and Verrucomicrobia (Figure 10). Other bacterial classes, including Actinobacteria, Coriobacteria, Melainabacteria, Deferribacteres, Saccharimonadia, Alphaproteobacteria, Deltaproteobacteria, Gammaproteobacteria and Mollicutes, were observed at lower abundance levels (i.e., <1%) in some (but not all) faecal samples. The relative abundance of bacteria from the class Bacteroidia was reduced in H rats (8.96 ± 2.2%) compared to the other groups (27.63 ± 2.2% to 36.46 ± 1.3%) (*p* < 0.0001). An increase in the relative abundance of bacteria from class Bacilli was observed in C rats (2.49 ± 1.0% to 4.08 ± 1.9%) compared to H rats (0.7 ± 0.3% to 0.75 ± 0.4%). A higher abundance of bacteria from the class Clostridia was observed for H and HGD rats, and this was more pronounced for H rats (45.03 ± 4.8% to 76.80 ± 4.9%) compared to C rats (37.81 ± 3.6% to 45.36 ± 3.5%) (*p* < 0.0001). Similarly, an increase in relative abundance of bacteria from the class Verrucomicrobiae was observed in HGD rats (21.16 ± 3.3%) compared to CGD rats (12.46 ± 3.6%), while a decrease in abundance was observed in H rats (4.47 ± 2.8%, *p* < 0.0001) compared to C rats (13.89 ± 2.0%).

**Figure 11:**
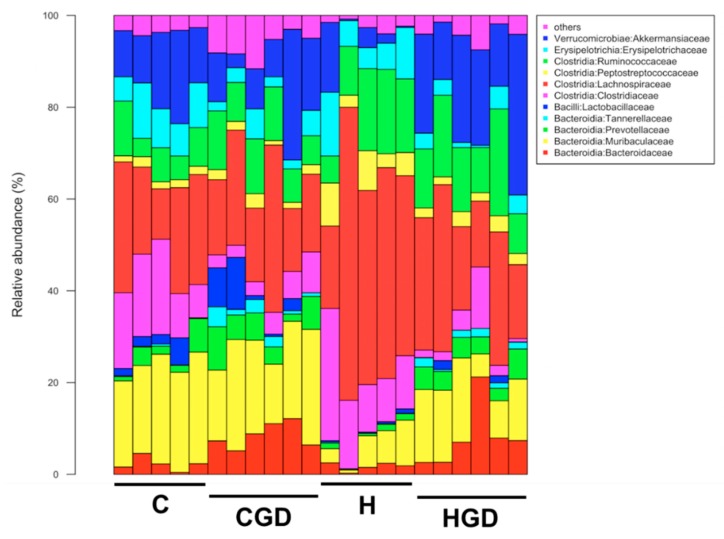
Taxonomic profiles of bacterial communities shown at the family level. C, corn starch diet-fed rats; CGD, corn starch diet-fed rats treated with *Garcinia dulcis* rind powder; H, high-carbohydrate, high-fat diet-fed rats; HGD, high-carbohydrate, high-fat diet-fed rats treated with *Garcinia dulcis* rind powder.

Analysis of the bacterial community structure at the family level showed that Bacteroidaceae (class Bacteriodia), Muribaculaceae (class Bacteriodia), Prevotellaceae (class Bacteroidia), Tannerellaceae (class Bacteroidia), Lachnospiraceae (class Clostridia), Lactobacillaeceae (class Bacilli), Clostridiaceae (class Clostridia), Peptostreptococcaceae (class Clostridia), Ruminococcaceae (class Clostridia), Erysipelotrichaceae (class Erysipelotricia) and Akkermansiacaeae (class Verrucomicrobia) were found to be most dominant in the faecal samples (Figure 11). The relative abundance of bacteria from family Ruminococcaceae was reduced for C rats (7.38 ± 1.4% to 9.75 ± 1.1%) compared to H rats (13.77 ± 2.4% to 14.39 ± 2.2%). A high abundance of bacteria from the family Lachnospiraceae was detected in H rats (41.85 ± 7.3%, *p* < 0.0001) compared to HGD rats (23.82 ± 3.6%) and C rats (20.78 ± 3.5% to 21.11 ± 2.9%). In contrast, the abundance of bacteria from families Muribaculaceae and Lactobacillaeceae was reduced in H rats (Muribaculaceae: 5.5 ± 1.6% to 12.8 ± 2.1%; Lactobacillaeceae: 0.4 ± 0.1% to 0.61 ± 0.4%) compared to C rats (Muribaculaceae: 19.9 ± 2.0% to 21.60 ± 1.2%, *p* < 0.05; Lactobacillaeceae: 2.34 ± 0.1% to 3.98 ± 0.2%) (Figure 11). Moreover, higher abundance of bacteria from family Bacteroidaceae was observed for both diets supplemented with *G. dulcis* rind powder (8.12 ± 2.8% to 8.49 ± 1.1%) compared to controls (1.72 ± 0.4% to 2.24 ± 0.7%).

**Figure 12:**
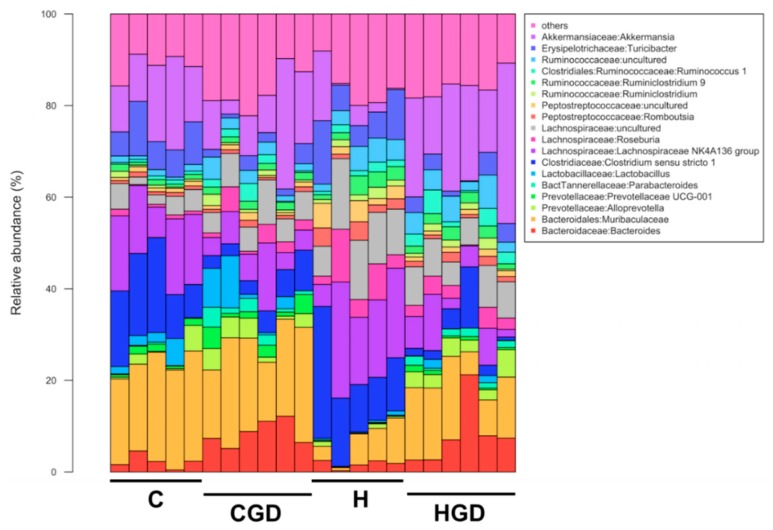
Taxonomic profiles of bacterial communities shown at the genus level. C, corn starch diet-fed rats; CGD, corn starch diet-fed rats treated with *Garcinia dulcis* rind powder; H, high-carbohydrate, high-fat diet-fed rats; HGD, high-carbohydrate, high-fat diet-fed rats treated with *Garcinia dulcis* rind powder.

Analysis of the bacterial community structure at the genus level showed that *Bacteroides* (family Bacteroidaceae), unclassified Muribaculaeceae, *Clostridium sensu stricto 1* (family Clostridiaceae), *Lachnospiraceae* NK4A136 group (family Lachnospiraceae), *Roseburia* (family Lachnospiraceae), *Ruminococcus 1* (family Ruminococcaceae), unclassified Ruminococcaceae, *Turicibacter* (family Erysipelotrichaceae) and *Akkermansia* (family Akkermansiaceae) were found to be most dominant in the faecal samples (Figure 12).

#### 2.5.5. Differentially Abundant zOTUs

Additionally, multivariate analysis of individual zOTUs using the R package Mvabund revealed that diet and treatment, as well as the interaction between diet and treatment changed the bacterial community structure in the faecal samples (Table 3).

A total of 19 zOTUs (1.70% of total zOTUs) belonging mostly to phylum Firmicutes were affected by diet (Table 3, Supplementary Table S1). One zOTU belonging to the phylum Actinobacteria was enriched in C rats (C and CGD rats), while bacteria belonging to the phylum Firmicutes and families Lachnospiraceae (genus: [*Bacteroides*] pectinophilus group, *Anaerostipes*, *Lachnospiraceae* NK4A136 group, *Lachnospiraceae* UCG-006, *Lachnospiraceae* UCG-008, *Roseburia*), Peptostreptococcaceae and Ruminococcaceae (genus: *Ruminiclostridium 9*) were either reduced or absent in C and CGD rats (Supplementary Table S1).

At the zOTU level, *G. dulcis* supplementation had a stronger effect than diet on the bacterial community structure by affecting the abundance of 35 zOTUs (3.14% of total zOTUs) (Table 3). zOTUs belonging to the phylum Firmicutes (families Clostridiaceae 1, Lachnospiraceae and Ruminococcaceae; genus *Clostridium sensu stricto 1*, *Acetitomaculum*, *Blautia*, *Lachnospiraceae* NK4A136 group, *Pygmaiobacter*, *Ruminococcaceae* UCG-010, *Ruminococcaceae* UCG-014 and UBA1819) were reduced in abundance or absent in CGD and HGD samples compared to C and H rats, respectively, while zOTU belonging to the families Lachnospiraceae (genus: unclassified) were enriched in the CGD and HGD rats (Supplementary Table S2). Bacteria belonging to the phylum Proteobacteria and family Parasutterella were enriched in CGD and HGD rats compared to C and H rats, while zOTUs belonging to the phylum Patescibacteria and family Saccharimonadaceae were enriched in the control samples (C and H) compared to samples from rats treated with *G. dulcis* rind powder (Supplementary Table S2).

#### 2.5.6. Multivariate Analysis of Physiological Data

A total of twenty-three physiological parameters were assessed and included in the analysis below (body weight, water intake, food intake, energy intake, feed efficiency, left ventricle and septum wet weight, right ventricle wet weight, liver wet weight, kidneys wet weight, spleen wet weight, retroperitoneal fat, omental fat, epididymal fat, total abdominal fat, plasma non-esterified fatty acids, plasma triglycerides, fat mass, lean mass, systolic blood pressure, oral glucose tolerance area under the curve, blood glucose concentrations at 120 min, plasma aspartate transaminase activity and plasma alanine transaminase activity) for rats fed with C and H diets and supplemented with *G. dulcis* rind powder.

Distance-based multivariate analysis shows that treatments have distinct responses on the physiological parameters. An overall effect of diet (Supplementary Figure S1, Supplementary Table S3; PERMANOVA, *p* = 0.0002) and treatment (Supplementary Figure S1, Supplementary Table S3; PERMANOVA, *p* = 0.0001) was observed on physiological states of rats.

There was statistical support for differences between C and H rats without *G. dulcis* rind powder (*p* = 0.0083) indicating an effect of basal diet on physiological parameters of rats. There was also an effect for the addition of *G. dulcis* to either of the diets (*p* = 0.0015 to 0.0023 for C and H, respectively, Supplementary Table S3), and these changes appear to be similar (Supplementary Figure S1). Rat physiological states in CGD group were more variable between replicates compared to C group (Supplementary Figure S1, Supplementary Table S3; PERMDISP; *p* = 0.0342) and H group (Supplementary Figure S1, Supplementary Table S3; PERMDISP; *p* = 0.0028).

#### 2.5.7. Correlation of Microbiota and Physiological Parameters

Combined analysis of bacterial community structure and physiological parameters were performed, and Mantel test showed that overall the bacterial community structure and the physiological data are correlated (Mantel statistic *r* = 0.3739; *p* = 0.0004). Among the physiological parameters, water intake, energy intake, feed efficiency, oral glucose tolerance test area under the curve, kidneys and liver weights and plasma triglycerides had the strongest correlation with bacterial community structure (Supplementary Figure S2).

Energy intake, oral glucose tolerance test area under the curve and systolic blood pressure correlated with the changes in the bacterial community structure for both C and H diet in response to *G. dulcis* supplementation. Total abdominal fat, energy intake, plasma non-esterified fatty acids, fat mass, retroperitoneal fat, epididymal fat, omental fat, body weight gain, feed efficiency, left ventricle wet weight, food intake, plasma triglycerides and kidney wet weight were correlated with the changes in the bacterial community structure for the C diet in response to the *G. dulcis* supplementation, while water intake was correlated with changes in the bacterial community structure for the H diet in response to the *G. dulcis* supplementation (Supplementary Table S4).

When physiological variables were further correlated with individual zOTUs, a total of 40 zOTUs were found to be correlated with at least 1 of the 17 physiological parameters (*p* < 0.05). 32 zOTUs belonged to the phylum Firmicutes, five zOTUs to the phylum Bacteroidetes, two zOTUs to the phylum Proteobacteria and one zOTU to the phylum Actinobacteria. Total abdominal fat (five of 40 zOTUs or 12.5%) and energy intake (seven of 40 zOTUs or 17.5%) were inversely correlated with the relative abundance of the selected zOTUs. Similarly, systolic blood pressure (one of 40 zOTUs or 2.5%) and oral glucose tolerance area under the curve (five of 40 zOTUs or 12.5%) were positively correlated with the selected zOTUs (Supplementary Table S5).

The relative abundances of zOTUs belonging to the phylum Firmicutes and the families Clostridiaceae 1 (genus *Clostridium sensu stricto 1*: zOTU3, zOTU1064), Lachnospiraceae (genus *Acetitomaculum*, *Anaerostipes*, *Blautia*, *Coprococcus* 2, *Lachnospiraceae* NK4A136 group, *Lachnospiraceae* UCG-006, *Lachnospiraceae* UCG-008, *Roseburia*, *Peptostreptococcaceae* and *Ruminococcaceae*: zOTU410, zOTU131, zOTU372, zOTU247, zOTU1122, zOTU400, zOTU156, zOTU118, zOTU162, zOTU70, zOTU166 and zOTU555) were inversely correlated with the physiological parameters included in this analysis, with the exception of water intake.

In contrast, several zOTUs belonging to the phylum Firmicutes (unclassified Lachnospiraceae) (zOTU304, zOTU469, zOTU473 and zOTU658) were positively correlated to the physiological parameters liver wet weight and oral glucose tolerance area under the curve (Supplementary Table S5).

Further, the relative abundances of bacteria belonging to phylum Actinobacteria and family Bifidobacteriaceae; phylum Proteobacteria and family Parasutterella; and phylum Bacteroidetes and family Tannerellaceae (for example: zOTU24, zOTU477, zOTU343, zOTU46, zOTU211, zOTU28, zOTU21 and zOTU230) were positively correlated to the physiological parameters including kidneys wet weight, liver wet weight, plasma triglycerides, plasma non-esterified fatty acids, oral glucose tolerance area under the curve and total abdominal fat (Supplementary Table S5).

## 3. Discussion

*Garcinia dulcis* is a tropical fruit used in Southeast Asia mainly for medicinal properties and occasionally as food, although the sour taste is a challenge in commercialising the fruit without processing [21]. *G. dulcis* fruit has a thick rind in proportion to the whole fruit, comparable to *G. mangostana* and *G. humilis* [20,22]. The rind and seed which form about 60% of the total fruit weight are rarely used and so these are regarded as waste. The rind, skin, seed and pomace contain bioactive compounds such as polyphenols, fibre, vitamins, enzymes and oils [23]. Hence, evidence for health benefits for these phytochemicals could assist commercialisation as well as reduce waste and the resulting environmental problems through decomposition.

Garcinol, morelloflavone and citric acid are the main phytochemical constituents of *G. dulcis* rind [24,25]. Garcinol is found in other *Garcinia* species such as *G. cambogia*, *G. bancana* and *G. indica* while citric acid is also common in species such as *G. cowa*, *G. indica*, *G. pedunculata* and *G. humilis* [20,26,27]. Hydroxycitric acid was not found in the rind, although this compound is common in other *Garcinia* species and has been proposed as an intervention for weight loss [28]. The doses of morelloflavone, garcinol and citric acid in this study in HGD rats were 5.4 mg/kg/day, 130 mg/kg/day and 480 mg/kg/day, respectively. These doses translate to an approximate intake of 52 mg/day, 1.26 g/day and 4.67 g/day of morelloflavone, garcinol and citric acid, respectively, in an adult human [29]. This translates to approximately 23 g of dried rind or 135 g of fresh rind, which is approximately the rind from two fresh fruits; these amounts suggest that partial purification may be necessary to produce a more concentrated nutraceutical product. Garcinol as the main polyisoprenylated benzophenone found in *G. dulcis* rind has been studied as an anti-cancer agent [30] while morelloflavone had beneficial effects on cardiovascular health [11]. Citric acid from *Garcinia* fruit could act synergistically with both garcinol and morelloflavone to improve cardiovascular parameters [20].

Although there are limited in vivo studies on the effects of *G. dulcis* rind and its constituents on metabolic syndrome, many studies discuss garcinol and its anti-cancer properties [12,31]. As an example, 0.05% garcinol in diet for 5 weeks reduced the incidence of colon carcinogenesis by 40.2% in rats with inflammation-induced colonic aberrant crypt foci [32]; this dose would correspond to about 25 mg/kg/day in an obese 500 g rat eating 25 g food/day. Garcinol showed its anti-cancer properties through its effects on inflammatory pathways, apoptotic pathways, epigenetic control, proliferation, angiogenesis and metastasis [30].

An in vitro study found that garcinol showed potential inhibition of lipid accumulation in adipocytes by dose-dependently inhibiting the cell population growth of 3T3-L1 cells [33]. In these preadipocytes, garcinol decreased C/EBPα and PPARγ expression, increased mRNA expression of adiponectin, and reduced expression of leptin, resistin and fatty acid synthase [33]. These findings could explain mechanisms responsible for the reduction of abdominal fat and adipocyte size reductions observed in our study. Administration of garcinol (25, 50 and 100 mg/kg/day) to streptozotocin-induced diabetic rats reduced concentrations of triglycerides, total cholesterol, LDL and VLDL while increasing HDL concentrations [34]. *G. dulcis* fruit extract reduced total cholesterol, triglyceride and LDL concentrations, and increased HDL concentrations [35]. Similarly, we found reduction of triglycerides and non-esterified fatty acids but without changes in total cholesterol concentrations.

The intake of 0.05% of garcinol per day in male F344 rats improved anti-oxidant and anti-inflammatory effects by decreasing expression of iNOS and COX-2 [32]. In addition, garcinol inhibited the generation of NO and O_2_^−^ species [32]. The inhibition of iNOS and COX-2 generation by garcinol was suggested due to the blocking of lipopolysaccharide-induced NF-κB production through suppression of IκBα and p38 mitogen-activated kinase [36]. These studies suggest that the changes in physiological and metabolic parameters observed in our study were due to a reduction in inflammatory and oxidative stress responses.

The present study showed an improvement of glucose tolerance and insulin response in HGD rats. Administration of 20 mg/kg/day of garcinol in streptozotocin-induced diabetic Wistar rats reduced fasting blood glucose concentrations [37]. Additionally, administration of 100 mg/kg/day of garcinol reduced HbA1c and blood glucose concentrations in streptozotocin-induced diabetic female Wistar rats [34].

In this study, we also found improvements in liver structure and function by decreased collagen deposition and reduced aspartate transaminase activity in HGD rats compared to H rats, similar to a previous study with *G. dulcis* fruit rind [8]. In addition, administration of 20 mg/kg/day of garcinol decreased aspartate transaminase and alanine transaminase activities compared to diabetic rats [37]. The reduction of liver fibrosis and collagen deposition by garcinol treatment was also noted here. Garcinol administration reduced aspartate transaminase activity and showed hepatoprotective and anti-fibrotic effects by reducing accumulation of extracellular matrix and expression of α-smooth muscle actin, TGF-β1 and Smad 2/3 proteins in dimethylnitrosamine-induced liver fibrosis in rats [38].

Treatment with *G. dulcis* rind powder showed cardioprotective effects in HGD rats. Administration of cyclodextrin-garcinol complex (20 mg/kg/day) in isoproterenol-induced cardiotoxicity showed marked cardioprotective activity through reduction of myocardial hypertrophy and concentrations of malondialdehyde and increased glutathione [39]. Garcinol (50 µmol/L for 24 h) inhibited the intrinsic histone acetyl-transferase activity of CBP/p300 and suppressed the expression of collagen I in fibroblasts [40], which could explain the reduction of collagen deposition in both heart and liver in HGD rats and the reduction in cardiac stiffness observed in our study.

Further, morelloflavone isolated from *G. dulcis* fruits has diuretic and hypotensive effects [41]. The combination of morelloflavone and 7-epiclusianone at a dose of 10.76 mg/rat and 42 mg/rat, respectively, from *G. brasiliensis* ethanol extract reduced body weight and liver weight compared to control high-fat diet [42]. Morelloflavone and 7-epiclusianone in the peel extract increased β-oxidation and decreased fatty acid synthesis to decrease steatosis and liver damage [42]. Morelloflavone counteracted restenosis by blocking injury-induced neointimal hyperplasia through the inhibition of vascular smooth muscle cell migration [43]. The inhibition of morelloflavone on vascular smooth muscle cell migration was due, in part, to its inhibition of the activation of migration-related kinases, including focal adhesion kinase, C-SRC, extracellular signal-related kinase and RhoA in vascular smooth muscle cells [43]. In isolated rat thoracic aorta, cumulative addition of 10^−9^ to 10^−5^ M of morelloflavone from *G. dulcis* dose-dependently relaxed noradrenaline-precontracted rat thoracic aorta [44]. In addition, the anti-atherogenic effects of morelloflavone from an extract of *G. dulcis* leaves at doses of 0.005% and 0.01% in hypercholesterolaemic rabbits were shown by reductions in plasma cholesterol and triglycerides and reduction in atheromatous lesions [10]. However, there are limited studies on the in vivo effects of morelloflavone in rodents.

Gut microbiota analysis showed marked changes on the structure of colonic bacterial communities in H rats compared to C rats; the communities of both C and H rats were changed by addition of *G. dulcis* rind powder to the diet. The gut microbiota in obese subjects in both human and high-fat diet-fed mice showed increased F/B ratio [45]. In this study, the F/B ratio was decreased in HGD rats compared to H rats similar to the finding from previous study using garcinol [46].

The decrease in relative abundance of class Bacteroidia in H rats agrees with a previous study using high-fat diet [47]. *G. dulcis* treatment reduced relative abundance of class Clostridia compared to H rats. The relative abundance of class Verrucomicrobiae and Bacteroidetes (Bacteroidia) was increased in HGD rats and C rats compared to H rats, similar to previous study showing the increase in this class when a high-fat diet was added with garcinol [46]. At family level, the addition of *G. dulcis* increased the population of Akkermansiaceae, Lachnospiraceae and Bacteroidaceae. This finding was also found in a previous study using garcinol as treatment in high-fat diet, except for Lachnospiraceae [46], suggesting that garcinol present in *G. dulcis* rind modulated the microbiota in H rats. At genus level, the increase in relative abundance of *Akkermansia* and *Bacteroides* in HGD rats was also previously found [46]. The multivariate analysis showed that zOTUs belonging to the phylum Firmicutes were reduced in their relative abundances in CGD and HGD rats, which explains the reduction of F/B ratio in these samples.

In this study, the bacterial community structures are found to correlate with individual physiological parameters. When compared with individual zOTU, *Bifidobacterium* was positively correlated with obesity-related parameters. However, *Bifidobacterium* was found to have protective effects against obesity in high-fat diet-fed rats [48]. Furthermore, *Bifidobacterium* and *Akkermansia* among others are more prevalent in individual with lean phenotypes while *Bacteroides*, *Anaerostipes* and *Ruminococcus* are more prevalent in obese individuals [49].

Furthermore, the genus *Lachnospiraceae* NK4A136 group was positively correlated with area under the curve for blood glucose concentration. Previous study showed that *Lachnospiraceae* was positively associated with diabetes [50]. Additionally, the abundance of *Bacteroides* and *Parabacteroides* was positively correlated while *Roseburia* was negatively correlated with fasting blood glucose and area under the curve in obese diabetic *ob/ob* mice fed with capsaicin [51].

The relative abundance of genus *Acetitomaculum* was positively correlated with normalised left ventricle and septum wet weight, which indicate reduced cardiac hypertrophy but *Anaerostipes*, *Lachnospiraceae* NK4A136 group and *Lachnospiraceae* UCG-008 were negatively correlated with this parameter. In animal studies, *Eubacteria*, *Anaeroplasma*, *Roseburia*, *Oscillospora* and *Dehalobacteria* were found to be active in preventing atherosclerosis, while the absence of microbiota could increase the formation of atherosclerotic lesion [52,53]. Other bacteria such as *Porphyromonas gingivalis* and *Aggregatibacter actinomycetemcomitans* increased the progression of atherosclerosis in an animal model [52].

Blood pressure is closely linked to the diversity, richness and evenness of the microbiota living in the gut and is affected by the F/B ratio [52]. Further, a gut metabolite trimethylamine *N*-oxide (TMAO) was identified as a major affecting factor in cardiovascular disease [52] with TMAO playing a role in the development of cardiac hypertrophy and cardiac fibrosis [54]. Other than TMAO, gut metabolites such as secondary bile acids, indoxyl sulphate, *p*-cresyl sulphate and phenylacetyl-glutamine can also influence cardiovascular disease risk [52].

Dysbiosis of the gut microbiota has been associated with non-alcoholic fatty liver disease [55]. Increased liver weight was correlated with the increased deposition of hepatic fat and inflammation [18]. Here, the genus *Parabacteroides* and the family Lachnospiraceae were positively associated with normalised liver wet weight but the genus *Clostridium sensu stricto 1* and *Acetitomaculum* were negatively associated. Analysis of the gut microbiota in non-alcoholic fatty liver disease patients showed a lower percentage of Bacteroides and higher amounts of Prevotella and Porphyromonas species compared to lean controls [56]. The decreases in Lachnospiraceae, Lactobacillaceae and Ruminococcaceae have been observed in patients with non-alcoholic fatty liver disease [57]. The change in gut microbiota promotes the development of non-alcoholic fatty liver disease by facilitating processes of inflammation, insulin resistance, bile acids and choline metabolism, changing intestinal permeability, production of ethanol in the intestine and interaction with innate immunity [55,58].

In this study, we did not find any abnormal changes in gastrointestinal or liver structures, which indicates that the doses given in this study were not toxic. The no-observed-adverse-effect level value for repeated dose toxicity for citric acid in rats was 1200 mg/kg/day [59], which is higher than the dose given in this study. Acute administration of garcinol was safe to use up to 2000 mg/kg body weight in Wistar rats [60]. Sub-chronic garcinol dosage for 90 days showed no-observed-adverse-effect level value of 100 mg/kg/day without changes in gross histopathological or other biochemical parameters [60], which is around two thirds of the dose in HGD rats. Further, repeated administration of 300 mg/kg of garcinol did not produce any signs of toxicity or increased mortality [34]. The LD_50_ in rats of garcinol was taken as 1000 mg/kg [34]. Administration of 8 mg/kg/day of morelloflavone in mice showed no observed toxicity [61]; this dose was higher than the dose given in this study of 5.4 mg/kg/day.

The markedly decreased body weight in treated rats can be associated with the reduction in food intake, so this could suggest that the combination of citric acid, garcinol and morelloflavone in *G. dulcis* rind triggers the appetite satiety mechanisms that inhibit food intake, although there are no studies on these responses. Possible mechanisms include similar responses as hydroxycitric acid which reduced appetite and body weight in human subjects [62], increased release of serotonin from rat brain cortex slices [63] and inhibited serotonin uptake [64].

## 4. Materials and Methods

### 4.1. *Garcinia dulcis* Rind, Pulp and Seed Preparation and Analyses

*G. dulcis* fruits were obtained from South Johnstone Research Facility (Centre for Wet Tropics Agriculture, Boogan, QLD, Australia). The fruits were then separated into rind, pulp and seed and weighed. The rind, pulp and seeds were frozen separately at −20 °C before freeze-drying and grinding into powder. Samples of rind, pulp and seed powder were analysed to identify the compounds present. The remaining powders were kept at 4 °C until further use. Analyses were performed on an Agilent 1200 series HPLC system consisting of a diode array detector (G4212B), binary pump (G4220A), an autosampler (G4226A), a vacuum degasser and a column oven. All solvents and reagents used were HPLC or analytical grade. For analysis of garcinol and morelloflavones, the column was linked to an Agilent 6130 single quadrupole mass spectrometer as detector (Agilent Technologies Australia, Mulgrave, VIC, Australia).

#### 4.1.1. Citric Acid Analysis

Analysis of organic acids was based on the 2016 United States Pharmacopeia (USP) *Garcinia* hydroxycitric acid method. All reagents and solvents were HPLC grade with Milli-Q water. Mobile phase was 0.136% (*w*/*v*) potassium dihydrogen phosphate in 3% phosphoric acid adjusted to pH 2.5. Briefly, about 250 mg of the extracts was weighed and extracted in 5 mL of 3% phosphoric acid with sonication for 15 min. After centrifuging for 5 min, an aliquot of the supernatant was taken up in a HPLC vial and run against reference standards of hydroxycitric acid calcium salt, citric acid and malic acid.

The analysis was performed using a Phenomenex 250 mm C18 column with 1 mL/min of isocratic mobile phase over 25 min. Reference standards were injected and prepared as a calibration curve for calculation of the hydroxycitric acid and citric acid concentrations. For the hydroxycitric acid measurements, the rind of *Garcinia quaesita* was held as a reference sample at Analytical Research Laboratory, Southern Cross University, Lismore, NSW, Australia for herbal authentication.

#### 4.1.2. Garcinol Analysis

The chromatography was performed with a Phenomenex Kinetex C18 HPLC column (100 × 4.6 mm) using a gradient method [65]. Dried ground rind, pulp and seed were quantitatively extracted by mixing 0.2 to 0.25 g with 25 mL of acetonitrile in a volumetric flask sonicated for 30 min. An aliquot of the solvent sample was centrifuged and 200 µL of the supernatant was taken into HPLC vial for analysis. The mobile phases were solvent A (0.1% formic acid, Milli-Q water) and solvent B (acetonitrile with 0.1% formic acid); optimal separations were obtained using a gradient of 60% to 95% B over 0–28 min, with flow rate of 0.75 mL/min.

Reference standard of garcinol (Sigma-Aldrich Australia, Castle Hill, NSW, Australia) was prepared in acetonitrile at 0.4 mg/mL, then diluted to produce a five-point calibration curve. Quantification was performed based on calibration curve of reference standards, peak area at 240 nm and sample dilution. Total benzophenones were calculated as garcinol and results were expressed as % *w*/*w*.

#### 4.1.3. Morelloflavone Analysis

The chromatography was performed on a Phenomenex luna C18 HPLC column (100 × 4.6 mm) using a gradient method of water and acetonitrile with 0.005% trifluoroacetic acid over 28 min. The solvent gradient for separation of target constituents started at 10% acetonitrile increased as a gradient to 95% acetonitrile over 18 min, at a flow rate of 0.75 mL/min and an injection volume of 5 µL. Morelloflavones are biflavones of naringin (flavonone) and luteolin (flavone) units, hence naringin was used as the reference standard for calculating the quantity of morelloflavones. Calibration standards of naringin (Sigma-Aldrich Australia) were prepared in water, at concentrations from 0.01 to 0.4 mg/L. Specific detection and calibration curves for each compound were performed at 280 nm. Quantification was performed using the Chemstation software (Agilent Technologies Australia) based on reference standards, peak area and sample dilution at a specific wavelength of 280 nm for each peak identified as a biflavonone or glycoside.

### 4.2. Rats and Diets

A total of 48 male Wistar rats (8–9 weeks old) were obtained from the Animal Resource Centre, Murdoch, WA, Australia. Rats were housed individually in a temperature-controlled room (22 ± 2 °C) under 12-h light/dark cycle environment at the University of Southern Queensland animal house with free access to food and water. All experimental protocols were approved by the University of Southern Queensland Animal Ethics Committee (project number: 16REA014; approved on 30 September 2016), which operates under the guidelines of the Australian National Health and Medical Research Council. Rats were allowed to adapt for a week and upon reaching 330–340 g body weight (337 ± 1 g body weight, *n* = 48), they were randomly allocated into 4 experimental groups (*n* = 12 per group) and fed with corn starch diet (C), corn starch diet with 5% (*w*/*w*) *G. dulcis* rind powder (CGD), high-carbohydrate, high-fat diet (H) or high-carbohydrate, high-fat diet with 5% (*w*/*w*) *G. dulcis* rind powder (HGD). Rats in C and CGD received corn starch diet for the first 8 weeks, and H and HGD groups received high-carbohydrate, high-fat diet for the first 8 weeks. C and H diet compositions have been previously reported; both contain about 68% carbohydrate as corn starch in the C diet and as condensed milk and fructose in the H diet with 24% fat in the H diet mainly as beef tallow compared to ~1% fat in the C diet [18]. *G. dulcis* rind powder was added to groups CGD and HGD for the last 8 weeks while C and H rats were kept on their respective diets without supplementation for the whole 16-week protocol. Moreover, H and HGD rats were supplied with 25% (*w*/*v*) fructose in drinking water. Body weight and intakes of water and food were recorded daily by weighing of rats individually, and their remaining food and water, and feed efficiency was calculated [18]. Daily energy intake was calculated from the daily food and water intakes during the last 8 week of protocol [18]. The % increase in body weight was calculated as the difference in body weight between week 8 and week 16.

### 4.3. Measurements in Living Rats

Oral glucose tolerance tests were conducted after overnight (12 h) food deprivation at weeks 8 and 16 with fructose water in H and HGD groups substituted with normal water. After measuring basal blood glucose concentrations in tail vein blood using glucometer (Freestyle lite, Abbot Diabetes Care, Doncaster, VIC, Australia), rats were given 2 g/kg glucose through 40% aqueous glucose solution by oral gavage and blood glucose concentrations were measured in tail vein blood at 30, 60, 90 and 120 min after glucose loading [18].

Insulin tolerance tests were performed at 8 and 16 weeks of protocol. Rats were deprived of food five hours prior to the procedure. Fructose water in H and HGD groups was replaced with normal drinking water. After measuring basal blood glucose concentrations in tail vein blood using glucometer (Freestyle lite, Abbot Diabetes Care, Doncaster, VIC, Australia), 0.33 IU/kg insulin (Humulin R, Eli Lilly Australia, West Ryde, NSW, Australia) was administered intraperitoneally. Glucose concentrations in tail vein blood samples were measured at 30, 60, 90 and 120 min following insulin administration. The rats were withdrawn from the protocol when plasma glucose concentrations dropped below 1.1 mmol/L and were immediately gavaged with 5 mL of 50% aqueous glucose solution.

Dual-energy X-ray absorptiometry measurements were performed on rats at 8 and 16 weeks using a Norland XR46 DXA instrument (Norland Corp., Fort Atkinson, WI, USA) [18]. Rats were anaesthetised using isoflurane (Lyppard Australia Ltd. Pty, Northgate, QLD, Australia).

Whole body metabolism was measured at 16-week using 4 chamber OxyMax system (Columbus Instruments, Columbus, OH, USA) by placing one rat per chamber. Rats had free access to food and water during the experiment. Carbon dioxide production (V_CO2_) and oxygen consumption (V_O2_) were determined from each chamber. Respiratory exchange ratio (V_CO2_/V_O2_) was quantified by OxyMax software (v. 4.86). Energy expenditure was quantified based on the exchange of oxygen for carbon dioxide that occurs during metabolism of food [66].

Systolic blood pressure was measured at 8 and 16 weeks with rats lightly sedated with isoflurane (Lyppard Australia Ltd. Pty). Measurements were performed using an MLT1010 Piezo-Electric Pulse Transducer (ADInstruments, Bella Vista, NSW, Australia) and an inflatable tail-cuff connected to an MLT844 Physiological Pressure Transducer (ADInstruments) connected to a PowerLab data acquisition unit (ADInstruments) [18].

### 4.4. Measurements after Euthanasia

The isolated Langendorff heart preparation evaluated left ventricular function in rats [18]. Intraperitoneal Lethabarb (pentobarbitone sodium, 100 mg/kg; Virbac, Peakhurst, NSW, Australia) was used to induce terminal euthanasia. Following euthanasia, heparin (~200 IU, Sigma-Aldrich Australia) was injected into the right femoral vein. Blood (~5 mL) was then drawn from the abdominal aorta and kept in heparinised tubes. Isovolumetric ventricular function was assessed by inserting a latex balloon catheter into the left ventricle of the isolated heart attached to a Capto SP844 MLT844 physiological pressure transducer and Chart software on a MacLab system (ADInstruments) [18]. Heparinised blood was centrifuged at 5000× *g* for 10 min within 30 min of collection. Plasma was stored at −20 °C. Plasma concentrations of triglycerides, non-esterified fatty acids and total cholesterol, and plasma activities of aspartate transaminase and alanine transaminase were measured using kits and standards supplied by Olympus (Tokyo, Japan) using an AU 400 Olympus analyser [18]. Plasma C-reactive protein concentrations were determined using a commercial ELISA kit (BD Bioscience, North Ryde, NSW, Australia).

Organ weights were measured for right and left ventricles, liver and retroperitoneal, epididymal and omental fat pads shortly after euthanasia. Retroperitoneal, epididymal and omental fat pads were included together as total abdominal fat. Organ weights were normalised relative to the tibial length at the time of their removal (in mg/mm) [18].

For histological analysis, heart, liver, ileum and colon portions were collected about 5–7 min after euthanasia and fixed in 10% neutral buffered formalin for 3 days. The samples were dehydrated using an automated tissue processor and embedded in paraffin wax. Thin sections (~5 µm) of tissues were cut and stained with haematoxylin and eosin or picrosirius red stain. Haematoxylin and eosin stained sections were examined using an EVOS FL Colour Imaging System (v 1.4 (Rev 26059); Advanced Microscopy Group, Bothell, WA, USA) to observe infiltration of inflammatory cells in liver and heart and for determining fat vacuoles in liver [18]. Left ventricular and liver sections were stained with picrosirius red to study collagen distribution using BX53 Olympus microscope (Olympus, Eagle Farm, QLD, Australia) [18].

About 0.5 cm^2^ of retroperitoneal adipose tissue was fixed in 10% neutral buffered formalin and kept at 4 °C for at least 72 h. The formalin was then discarded and replaced with 70% ethanol solution. Tissue was sectioned about 10 µm thick and stained with haematoxylin and eosin stain [67]. Sectioned slides were observed using EVOS FL Colour Imaging System to observe deposition of fat. Mean adipocyte area and adipocyte area distribution were calculated using ImageJ [68] and Prism Version 6.00.

Small liver portions were embedded in Tissue-Tek O.C.T. Compound (ProSciTech, Kirwan, QLD, Australia) and stored in −20 °C. Tissue was sectioned using a cryostat (10 µm), air-dried and stained using Oil red O stain. Stained sections were observed using EVOS FL Colour Imaging System to observe deposition of fat. Mean liver fat vacuoles area was calculated using ImageJ [68]. Liver glycogen and plasma catalase activity were measured using published methods with modifications [69,70].

Faecal samples for microbiota analysis were collected from the colon using clean forceps after euthanasia and stored in Eppendorf tubes at −80 °C. Faecal samples for lipid content analysis were collected from the cage on the morning of euthanasia and stored in Eppendorf tubes at −20 °C [71]. For lipid analysis, 1 g of faeces were air-dried and homogenised in 5 mL saline solution. The homogenisation process produced a suspension that was mixed with 5 mL of 2:1 (*v*/*v*) chloroform:methanol mixture and centrifuged at 1000× *g* for 10 min. After centrifugation, the lower liquid phase was separated and transferred into a pre-weighed test tube. The lipids were air-dried and weighed.

### 4.5. Gut Microbiota Analysis

Gut microbiota diversity profiling was performed based on 16S rRNA gene sequencing on rat faecal samples collected at euthanasia and stored at −80 °C.

#### 4.5.1. DNA Extraction of Microbial Samples

Total microbial community DNA was extracted from faecal samples using the DNeasy Powersoil Kit (Qiagen Australia, Chadstone, VIC, Australia) following the manufacturer’s instructions and protocol published previously [72].

#### 4.5.2. 16S rRNA Gene Amplification and Sequencing

Bacterial communities from faecal samples were investigated by sequencing 16S rRNA gene amplicons. Below mentioned primers, *341F* and *785R* were used to amplify the V3-V4 regions of the 16S rRNA gene.

*341F* (TCGTCGGCAGCGTCAGATGTGTATAAGAGACAGCCTACGGGNGGCWGCAG)
*785R* (GTCTCGTGGGCTCGGAGATGTGTATAAGAGACAGGACTACHVGGGTATCTAATCC)

The reaction mixture (50 μL total volume per sample) consisted of Econotaq^®^ PLUS GREEN 2× Master Mix (Astral Scientific, Gymea, NSW, Australia) (25 μL), Ambion^®^ nuclease-free water (17 μL), the primer pair *341F* and *785R* (1.5 μL of each; 10 μM) and DNA template (5 μL). The PCR program consisted of an initial denaturation at 94 °C (2 min), followed by 35 cycles of denaturation at 94 °C (30 s), annealing at 55 °C (30 s) and extension at 72 °C (40 s) and a final extension of 72 °C (7 min). PCR products were then quantified using gel electrophoresis. Paired-end sequencing (2 × 300 bp) of the resulting 16S rRNA gene amplicons was performed at the Ramaciotti Centre for Genomics, University of New South Wales on an Illumina MiSeq platform as per the MiSeq System User Guide [73].

#### 4.5.3. 16S rRNA Gene Sequencing Analysis

Sequence data were initially quality-filtered and trimmed using TRIMMOMATIC version 0.36 truncating reads if the quality dropped below 20 in a sliding window of 4 bp [74]. USEARCH version 11.0.667 [75] was used for further processing [76] to merge and quality-filter sequencing reads, excluding reads with <250 or >550 nucleotides, in addition to reads with more than one ambiguous base or an expected error of more than 1. Filtered sequences were denoised and clustered into unique sequences (zOTUs) using the UNOISE algorithm [77] implemented in USEARCH. zOTU represent unique bacterial entities and are roughly equivalent to species or strains. Chimeric sequences were removed de novo during clustering and subsequently in reference mode using UCHIME [78] with the SILVA database (https://www.arb-silva.de/browser/) (SILVA SSURef 132 NR) as a reference [79]. zOTUs were then taxonomically classified (i.e., assigned a likely taxonomic name) by BLASTN [80] against the SILVA database. All non-bacterial OTUs were removed along with non-BLAST aligned and singleton zOTUs. Finally, processed sequences were mapped on zOTU sequences to calculate the distribution and counts of each zOTU in every sample. Only zOTUs occurring in more than two samples were considered for further statistical analysis.

### 4.6. Statistical Analysis

#### 4.6.1. Physiological and Metabolic Parameters

These data are presented as mean ± standard error of the mean (SEM). Data from C, CGD, H and HGD groups were assessed by two-way analysis of variance. When interaction and/or the main effects were significant, means were compared using Newman–Keuls multiple comparison post hoc test. *p* value of <0.05 was defined as statistically significant. All statistical analyses were performed using Prism version 6.00 for Windows (GraphPad Software, San Diego, CA, USA).

#### 4.6.2. Microbiota Community Analysis

Rarefaction curves were generated using the *rarecurve* function in *vegan* [81] and used to determine if a sufficient representation of the sample’s microbiota had been achieved given the sequencing effort. Prior to further analysis, the numbers of sequences were standardised across samples to account for different sequencing depths by randomly subsampling each sample to the lowest number of sequences counts obtained for any given sample (i.e., 14,908 counts). Bacterial alpha-diversities (i.e., zOTU richness and Shannon’s diversity) were calculated in R (version 3.5.3) using the *rrarefy* function in the *vegan* package for community ecology analysis [82]. A one-way analysis of variance test in GraphPad Prism 8.0.2 (San Diego, CA, USA) followed by Tukey’s pairwise comparisons test was used to determine the significance between the different groups, a *p* value <0.05 was considered to be significant.

For multivariate analysis of bacterial communities, zOTU table was imported into PRIMER [83] to compare the community structure (i.e., relative abundance data). Bray–Curtis similarity coefficients were calculated using square-root transformed zOTU abundances, and the resulting similarity matrix was visualised using nMDS. PERMANOVA [84] with 9999 random mutations was used to test the effect of diet and treatment on bacterial communities in rat faecal samples.

For multivariate analysis of physiological data, the table of physiological variables was imported into PRIMER [83]. Euclidean distance matrix coefficients were calculated using physiological data, and the resulting similarity matrix was visualised using nMDS. PERMANOVA [84] with 9999 random mutations was used to test the effect of diet and treatment on the physiological data in rat faecal samples.

To determine which microbial zOTUs were affected by diet, treatment and the interaction of the two factors, a two-factor design was used to adjust the data to a Multivariate Generalised Linear Model (MGLM) using the multivariate package MVabund [85]. Each OTU was treated as a variable fitted to a separate Generalised Linear Model (GLM) using a negative binomial distribution. An adjusted *p* value of <0.05 was considered to be significant.

#### 4.6.3. Correlation Analysis

The physiological parameters that contributed the most to the differences in the bacterial community structure were investigated using the functions *envfit* and *bioenv* in the R package *vegan*. Moreover, a correlation analysis was carried out using the *rcorr* function in the *Hmisc* package. A Pearson correlation coefficient was used to identify the individual zOTUs that were linearly correlated with the physiological parameters.

## 5. Conclusions

*G. dulcis* rind as a waste product from a widespread tropical fruit has potential to be developed as a source of nutraceuticals including garcinol, morelloflavone and citric acid to attenuate metabolic syndrome, especially obesity, by improving diet-induced cardiovascular, liver, metabolic and gut microbiota changes. We suggest that the observed effects were due to the additive effects of garcinol, citric acid and morelloflavone. A combination of anti-inflammatory and anti-oxidant responses with appetite suppression allows multiple symptoms to be targeted. Hence, further studies should be conducted to find feasible methods to store and then process the fruit rind, and to extract the phytochemicals from the fruit rind to produce sustainable, low-cost interventions for metabolic syndrome [86].

## Figures and Tables

**Table 1:** Effects of *Garcinia dulcis* rind on physiological, metabolic and cardiovascular parameters.

Variables	C	CGD	H	HGD	*p* Value		
					Diet	Treatment	Interaction
**Physiological Parameters**							
Initial body weight, g	338 ± 0.9^a^	339 ± 0.7^a^	337 ± 0.7^a^	337 ± 0.7^a^	0.35	0.46	0.32
8-week body weight, g	355 ± 6.6^b^	362 ± 7.0^b^	423 ± 6.4^a^	422 ± 10^a^	<0.0001	0.75	0.61
16-week body weight, g	404 ± 8^b^	306 ± 6^d^	508 ± 11^a^	362 ± 10^c^	<0.0001	<0.0001	0.011
Food intake, g/day	41.2 ± 2.3^a^	26.3 ± 2.3^b^	27.0 ± 2.5^b^	20.1 ± 1.9^b^	0.0001	<0.0001	0.10
Water intake, g/day	23.6 ± 2.6^a^	28.7 ± 2.9^a^	28.6 ± 6.0^a^	37.6 ± 4.5^a^	0.10	0.09	0.64
Garcinol intake, mg/kg/day	-	210 ± 7	-	130 ± 8	-	-	-
Citric acid intake, mg/kg/day	-	780 ± 27	-	480 ± 23	-	-	-
Morelloflavone intake, mg/kg/day	-	7.3 ± 0.2	-	5.4 ± 0.1	-	-	-
Energy intake, kJ/day	462 ± 28^b^	318 ± 10^c^	603 ± 52^a^	501 ± 12^b^	<0.0001	0.0002	0.49
Feed efficiency (8–16 weeks), g/kJ	0.10 ± 0.01^a^	−0.18 ± 0.02^b^	0.15 ± 0.02^a^	−0.11 ± 0.02^b^	0.002	<0.0001	0.58
Body weight gained (8–16 weeks), %	13.7 ± 1.0^b^	−15.4 ± 0.8^c^	20.1 ± 2.8^a^	−13.0 ± 2.2^c^	0.024	<0.0001	0.29
Abdominal circumference 8 weeks, cm	16.1 ± 0.2^b^	16.6 ± 0.3^b^	19.8 ± 0.5^a^	20.4 ± 0.5^a^	<0.0001	0.18	0.91
Abdominal circumference 16 weeks, cm	18.4 ± 0.4^b^	15.0 ± 0.2^d^	22.6 ± 0.4^a^	16.8 ± 0.4^c^	<0.0001	<0.0001	0.001
Whole body lean mass 8 weeks, g	283 ± 12^a^	300 ± 4^a^	310 ± 6^a^	302 ± 5^a^	0.047	0.56	0.09
Whole body lean mass 16 weeks, g	289 ± 10^a^	267 ± 5^a^	297 ± 8^a^	265 ± 13^a^	0.71	0.007	0.62
Whole body fat mass 8 weeks, g	57 ± 5^b^	51 ± 8^b^	94 ± 8^a^	105 ± 19^a^	0.002	0.86	0.55
Whole body fat mass 16 weeks, g	96 ± 9^b^	23 ± 3^c^	203 ± 12^a^	84 ± 6^b^	<0.0001	<0.0001	0.012
Bone mineral content 8 weeks, g	11.0 ± 0.6^a^	10.6 ± 0.5^a^	11.8 ± 0.3^a^	12.0 ± 0.6^a^	0.025	0.83	0.81
Bone mineral content 16 weeks, g	12.4 ± 0.5^b^	10.0 ± 0.2^c^	16.1 ± 0.5^a^	11.8 ± 0.3^b^	<0.0001	<0.0001	0.019
Bone mineral density 8 weeks, g/cm^2^	0.163 ± 0.004^a^	0.160 ± 0.005^a^	0.168 ± 0.004^a^	0.164 ± 0.002^a^	0.26	0.39	0.87
Bone mineral density 16 weeks, g/cm^2^	0.173 ± 0.004^b^	0.165 ± 0.003^b^	0.185 ± 0.003^a^	0.162 ± 0.003^b^	<0.0001	0.18	0.027
Body mass index 16 weeks, g/cm^2^	0.63 ± 0.02^b^	0.54 ± 0.02^c^	0.77 ± 0.02^a^	0.60 ± 0.02^b^	<0.0001	<0.0001	0.052
Retroperitoneal fat, mg/mm tibial length	255 ± 22^b^	71 ± 7^d^	497 ± 33^a^	160 ± 18^c^	<0.0001	<0.0001	0.001
Epididymal fat, mg/mm tibial length	101 ± 16^b^	39 ± 6^c^	165 ± 18^a^	49 ± 5^c^	0.006	<0.0001	0.040
Omental fat, mg/mm tibial length	159 ± 14^b^	66 ± 4^d^	260 ± 11^a^	115 ± 8^c^	<0.0001	<0.0001	0.015
Total abdominal fat, mg/mm tibial length	515 ± 45^b^	176 ± 13^d^	951 ± 49^a^	325 ± 29^c^	<0.0001	<0.0001	0.001
Visceral adiposity index, %	5.84 ± 0.43^b^	2.60 ± 0.20^d^	8.65 ± 0.39^a^	4.24 ± 0.37^c^	<0.0001	<0.0001	0.11
Kidney wet weight, mg/mm tibial length	50.2 ± 1.2^b^	40.7 ± 1.2^c^	56.0 ± 1.6^a^	47.1 ± 1.3^b^	<0.0001	<0.0001	0.82
Heat production 16 weeks, kcal/hour	4.06 ± 0.40^a^	2.06 ± 0.14^b^	4.20 ± 0.49^a^	3.59 ± 0.31^a^	0.029	0.003	0.06
Heat production area under the curve 16 weeks, (kcal/hour) × minutes	2878 ± 212^ab^	1419 ± 76^c^	3193 ± 97^a^	2500 ± 201^b^	0.002	<0.0001	0.053
Respiratory exchange ratio 16 weeks	0.973 ± 0.010^a^	0.986 ± 0.024^a^	0.886 ± 0.017^b^	0.898 ± 0.037^b^	0.012	0.66	0.99
Respiratory exchange ratio area under the curve 16 weeks	700 ± 9^a^	707 ± 16^a^	618 ± 15^b^	631 ± 18^b^	0.0005	0.48	0.82
Mean liver fat vacuoles area, µm^2^	14.1 ± 0.8^c^	9.8 ± 0.5^c^	150.1 ± 13.6^a^	73.0 ± 8.8^b^	<0.0001	<0.0001	<0.0001
Mean retroperitoneal adipocyte area, µm^2^	4299 ± 120^b^	2290 ± 67^c^	9966 ± 195^a^	4115 ± 326^b^	<0.0001	<0.0001	<0.0001
**Plasma Biochemistry and Glucose Response**							
Plasma alanine transaminase activity, U/L	28.4 ± 4.0^b^	33.8 ± 2.7^a^	42.3 ± 5.0^a^	39.1 ± 5.1^a^	0.025	0.89	0.28
Plasma aspartate transaminase activity, U/L	83.0 ± 8.0^b^	86.5 ± 9.0^b^	173.9 ± 24.0^a^	91.4 ± 6.6^b^	0.001	0.007	0.003
Plasma total cholesterol, mmol/L	1.37 ± 0.07^a^	0.95 ± 0.06^b^	1.66 ± 0.10^a^	1.50 ± 0.12^a^	<0.0001	0.003	0.16
Plasma triglycerides, mmol/L	0.45 ± 0.05^b^	0.25± 0.02^b^	1.15 ± 0.17^a^	0.35 ± 0.08^b^	0.0008	<0.0001	0.009
Plasma non-esterified fatty acids, mmol/L	0.87 ± 0.18^b^	0.35 ± 0.04^c^	3.25 ± 0.17^a^	0.80 ± 0.16^b^	<0.0001	<0.0001	<0.0001
Basal blood glucose 8 weeks, mmol/L	2.4 ± 0.1^b^	2.3 ± 0.1^b^	3.1 ± 0.1^a^	3.0 ± 0.1^a^	<0.0001	0.33	1.0
Basal blood glucose 16 weeks, mmol/L	2.6 ± 0.1^ab^	2.4 ± 0.2^b^	3.0 ± 0.1^a^	2.8 ± 0.1^ab^	0.003	0.13	1.0
Blood glucose area under the curve 8 weeks, mmol/L × minutes	488 ± 9^b^	494 ± 11^b^	596 ± 13^a^	585 ± 7^a^	<0.0001	0.82	0.43
Blood glucose area under the curve 16 weeks, mmol/L × minutes	466 ± 17^c^	401 ± 17^d^	602 ± 24^a^	544 ± 19^b^	<0.0001	0.003	0.86
Insulin response area under the curve 8 weeks, mmol/L × minutes	175 ± 23^b^	200 ± 34^b^	416 ± 55^a^	413 ± 12^a^	<0.0001	0.78	0.72
Insulin response area under the curve 16 weeks, mmol/L × minutes	149 ± 19^c^	154 ± 22^c^	373 ± 24^a^	281 ± 21^b^	<0.0001	0.028	0.017
Liver wet weight, mg/mm tibial length	231 ± 8^b^	212 ± 7^b^	358 ± 15^a^	341 ± 13^a^	<0.0001	0.14	0.91
Liver glycogen, mg/g	12.9 ± 0.4^a^	4.8 ± 0.5^c^	13.9 ± 0.4^a^	8.4 ± 1.2^b^	0.002	<0.0001	0.08
Plasma catalase activity, kU/L	39.0 ± 4.7^b^	45.6 ± 6.0^ab^	56.9 ± 8.1^a^	56.9 ± 8.8^a^	0.030	0.69	0.69
Plasma C-reactive protein, µg/mL	432 ± 5^b^	355 ± 22^c^	506 ± 8^a^	376 ± 19^c^	0.034	0.0002	0.19
Faecal lipid content, mg/g	21.8 ± 1.5^b^	8.1 ± 0.4^c^	43.3 ± 1.6^a^	42.1 ± 5.4^a^	<0.0001	0.015	0.039
**Cardiovascular Variables**							
Systolic blood pressure 8 weeks, mmHg	116 ± 3^b^	121 ± 2^b^	133 ± 4^a^	132 ± 3^a^	<0.0001	0.45	0.24
Systolic blood pressure 16 weeks, mmHg	117 ± 2^bc^	111 ± 3^c^	135 ± 2^a^	121 ± 5^b^	<0.0001	0.006	0.43
Left ventricle + septum wet weight, mg/mm tibial length	23.3 ± 1.4^a^	18.9 ± 0.7^a^	22.8 ± 1.4^a^	18.5 ± 0.8^a^	0.72	0.0005	0.99
Right ventricle wet weight, mg/mm tibial length	4.1 ± 0.2^ab^	3.6 ± 0.3^b^	4.7 ± 0.2^a^	4.0 ± 0.3^ab^	0.027	0.059	0.74
Left ventricular diastolic stiffness (κ)	21.6 ± 0.2^b^	21.9 ± 0.9^b^	26.7 ± 0.6^a^	22.5 ± 0.6^b^	<0.0001	0.004	0.001

Values are expressed as mean ± SEM, *n* = 8–12. Means with different superscripts (a, b, c or d) differ, *p* < 0.05. C, corn starch diet-fed rats; CGD, corn starch diet-fed rats supplemented with 5% *Garcinia dulcis* rind powder; H, high-carbohydrate, high-fat diet-fed rats; HGD, high-carbohydrate, high-fat diet-fed rats supplemented with 5% *Garcinia dulcis* rind powder.

**Table 2:** PERMANOVAs based on Bray–Curtis similarity measure for square-root transformed abundances of all rat faecal samples.

**Source**	**df**	**SS**	**MS**	**Pseudo-F**	***p* (perm)**	**Unique Perms**
Diet	1	6986.2	6986.2	7.0877	0.0001	9879
Treatment	1	7162.9	7162.9	7.267	0.0001	9905
Diet × treatment	1	2735.8	2735.8	2.7756	0.0001	9859
Res	18	17,742	985.68			
Total	21	34,411				
**PAIR-WISE TESTS**						
**Groups**				***t***	***p* (perm)**	**Unique Perms**
C, CGD				2.2169	0.0019	462
C, H				2.6651	0.0074	126
C, HGD				2.2135	0.0026	462
CGD, H				3.2027	0.0017	462
CGD, HGD				1.7959	0.0018	461
H, HGD				2.2624	0.0024	461
**PERMDISP (PAIRWISE COMPARISONS)**						
**Groups**					***t***	***p* (perm)**
C, CGD					0.28079	0.8116
C, H					1.6414	0.2101
C, HGD					1.6237	0.2481
CGD, H					2.4479	0.074
CGD, HGD					1.7921	0.1642
H, HGD					2.7484	0.0338

*p* values were calculated using 9999 permutations under a residual model. C, corn starch diet-fed rats; CGD, corn starch diet-fed rats treated with *Garcinia dulcis* rind powder; H, high-carbohydrate, high-fat diet-fed rats; HGD, high-carbohydrate, high-fat diet-fed rats treated with *Garcinia dulcis* rind powder.

**Table 3:** Summary of statistical tests on differential zOTU abundance.

**Global Test (GLMs) by Mvabund**		
Diet	*p* < 0.0001	
Treatment	*p* < 0.0001	
Diet × Treatment	*p* < 0.0001	
**Univariate Analysis by mvabund (*p* < 0.05)**		
**Factor**	**Number of Differentially Abundant OTUs**	**% of Total Number of OTUs**
Diet	19	1.70%
Treatment	35	3.14%
Diet × Treatment	2	0.18%
Total (unique zOTUs affected by one or more factors)	56	5.03%

## Supplementary Materials

Supplementary materials can be found at https://www.mdpi.com/1422-0067/21/1/272/s1. Table S1: Relative abundance of zOTUs affected by diet (ANOVA with *p* adjusted <0.05) between C, CGD, H and HGD rats; Table S2: Relative abundance of zOTUs affected by treatment (ANOVA with *p* adjusted <0.05) between C, CGD, H and HGD rats; Table S3: PERMANOVAs based on Euclidean distance matrix for square-root-transformed physiological data of all rat faecal samples; Table S4: Correlation between bacterial community structure and physiological parameters (*p* < 0.05); Table S5: Taxonomic assignments of the OTUs strongly correlated with physiological parameters; Figure S1: nMDS plot of physiological data from 23 physiological parameters, Figure S2: Correlation between bacterial community structure and environmental variables.

## Abbreviations

C	Corn starch diet-fed rats
CGD	Corn starch diet-fed rats supplemented with *Garcinia dulcis* rind powder
F/B	Ratio of Firmicutes/Bacteriodetes
H	High-carbohydrate, high-fat diet-fed rats
HGD	High-carbohydrate, high-fat diet-fed rats supplemented with *Garcinia dulcis* rind powder
GLM	Generalised linear model
HPLC	High-performance liquid chromatography
MGLM	Multivariate generalised linear model
nMDS	Non-metric, multi-dimensional scaling
PERMANOVA	Permutational multivariate analysis of variance
SEM	Standard error of the mean
TMAO	Trimethylamine N-oxide
USP	United States Pharmacopeia
zOTU	Zero-radius operational taxonomic unit

## References

[1] John O.D., Brown L., Panchal S.K., Ullah M., Ahmad A. (2019). Garcinia fruits: Their potential to combat metabolic syndrome. Nutraceuticals and Natural Product Derivatives: Disease Prevention & Drug Discovery.

[2] Lim T. (2012). Garcinia dulcis. Edible Medicinal and Non-Medicinal Plants.

[3] Cooper W. (2013). A taxonomic revision of *Garcinia* L.(Clusiaceae) in Australia, including four new species from tropical Queensland. Austrobaileya.

[4] Allen G. (2004). Mangosteen and its relatives; Sub-Tropical Fruit Club of Qld Inc. http://stfc.org.au/mangosteen-and-its-relatives.

[5] Kraikruan W., Klaipook W., Thanumthat R. (2017). Benefits of local humid tropical fruit trees in Thailand. Acta Hortic..

[6] Tuansulong K.A., Hutadilok-Towatana N., Mahabusarakam W., Pinkaew D., Fujise K. (2011). Morelloflavone from *Garcinia dulcis* as a novel biflavonoid inhibitor of HMG-CoA reductase. Phytother. Res..

[7] Abu Bakar M.F., Ahmad N.E., Suleiman M., Rahmat A., Isha A. (2015). *Garcinia dulcis* fruit extract induced cytotoxicity and apoptosis in HepG2 liver cancer cell line. BioMed Res. Int..

[8] Gogoi N., Gogoi A., Neog B., Baruah D., Singh K.D. (2017). Evaluation of antioxidant and hepatoprotective activity of fruit rind extract of *Garcinia dulcis* (Roxburgh) Kurz. Pharmacogn. Res..

[9] Khamthong N., Hutadilok-Towatana N. (2017). Phytoconstituents and biological activities of *Garcinia dulcis* (Clusiaceae): A review. Nat. Prod. Commun..

[10] Decha-Dier U., Hutadilok-Towatana N., Mahabusarakam W., Sawangjaroen K., Pinkaew D. (2008). Anti-altherogenic effects of morelloflavone from *Garcinia dulcis* leaves in cholesterol fed rabbits. J. Nat. Remed..

[11] Pinkaew D., Hutadilok-Towatana N., Teng B.-B., Mahabusarakam W., Fujise K. (2012). Morelloflavone, a biflavonoid inhibitor of migration-related kinases, ameliorates atherosclerosis in mice. Am. J. Physiol. Heart Circ. Physiol..

[12] Liu C., Ho P.C.-L., Wong F.C., Sethi G., Wang L.Z., Goh B.C. (2015). Garcinol: Current status of its anti-oxidative, anti-inflammatory and anti-cancer effects. Cancer Lett..

[13] Eckel R.H., Grundy S.M., Zimmet P.Z. (2005). The metabolic syndrome. Lancet.

[14] Grundy S.M. (2016). Metabolic syndrome update. Trends Cardiovasc. Med..

[15] Álvarez-Mercado A.I., Navarro-Oliveros M., Robles-Sánchez C., Plaza-Díaz J., Sáez-Lara M.J., Muñoz-Quezada S., Fontana L., Abadía-Molina F. (2019). Microbial population changes and their relationship with human health and disease. Microorganisms.

[16] Tian Y., Su L., Wang J., Duan X., Jiang X. (2018). Fruit and vegetable consumption and risk of the metabolic syndrome: A meta-analysis. Public Health Nutr..

[17] Rizzo N.S., Sabaté J., Jaceldo-Siegl K., Fraser G.E. (2011). Vegetarian dietary patterns are associated with a lower risk of metabolic syndrome: The adventist health study 2. Diabetes Care.

[18] Panchal S.K., Poudyal H., Iyer A., Nazer R., Alam A., Diwan V., Kauter K., Sernia C., Campbell F., Ward L. (2011). High-carbohydrate, high-fat diet–induced metabolic syndrome and cardiovascular remodeling in rats. J. Cardiovasc. Pharmacol..

[19] Arazo M., Bello A., Rastrelli L., Montelier M., Delgado L., Panfet C. (2011). Antioxidant properties of pulp and peel of yellow mangosteenfruits. Emirates J. Food Agric..

[20] John O., Wanyonyi S., Mouatt P., Panchal S., Brown L. (2018). Achacha (*Garcinia humilis*) rind improves cardiovascular function in rats with diet-induced metabolic syndrome. Nutrients.

[21] Subhadrabandhu S. (2001). Under-Utilized Tropical Fruits of Thailand. FAO Regional Office for Asia and the Pacific. http://www.fao.org/3/a-ab777e.pdf.

[22] Chaovanalikit A., Mingmuang A., Kitbunluewit T., Choldumrongkool N., Sondee J., Chupratum S. (2012). Anthocyanin and total phenolics content of mangosteen and effect of processing on the quality of mangosteen products. Int. Food Res. J..

[23] Sagar N.A., Pareek S., Sharma S., Yahia E.M., Lobo M.G. (2018). Fruit and vegetable waste: Bioactive compounds, their extraction, and possible utilization. Compr. Rev. Food Sci. Food Saf..

[24] Deachathai S., Mahabusarakam W., Phongpaichit S., Taylor W. (2005). Phenolic compounds from the fruit of *Garcinia dulcis*. Phytochemistry.

[25] Martin F.W., Campbell C.W., Ruberté R.M. (1987). Perennial Edible Fruits of the Tropics: An Inventory.

[26] Jena B.S., Jayaprakasha G.K., Sakariah K.K. (2002). Organic acids from leaves, fruits, and rinds of *Garcinia cowa*. J. Agric. Food Chem..

[27] Hemshekhar M., Sunitha K., Sebasatin Santhosh M., Devaraja S., Kemparaju K., Vishwanath B.S., Niranjana S.R., Girish K.S. (2011). An overview on genus *Garcinia*: Phytochemical and therapeutical aspects. Phytochem. Rev..

[28] Onakpoya I., Hung S.K., Perry R., Wider B., Ernst E. (2011). The use of *Garcinia* extract (hydroxycitric acid) as a weight loss supplement: A systematic review and meta-analysis of randomised clinical trials. J. Obes..

[29] Reagan-Shaw S., Nihal M., Ahmad N. (2008). Dose translation from animal to human studies revisited. FASEB J..

[30] Saadat N., Gupta S.V. (2012). Potential role of garcinol as an anticancer agent. J. Oncol..

[31] Behera A.K., Swamy M.M., Natesh N., Kundu T.K. (2016). Garcinol and its role in chronic diseases. Adv. Exp. Med. Biol..

[32] Tanaka T., Kohno H., Shimada R., Kagami S., Yamaguchi F., Kataoka S., Ariga T., Murakami A., Koshimizu K., Ohigashi H. (2000). Prevention of colonic aberrant crypt foci by dietary feeding of garcinol in male F344 rats. Carcinogenesis.

[33] Hsu C.L., Lin Y.J., Ho C.T., Yen G.C. (2012). Inhibitory effects of garcinol and pterostilbene on cell proliferation and adipogenesis in 3T3-L1 cells. Food Funct..

[34] Mali K.K., Dias R.J., Havaldar V.D., Yadav S.J. (2017). Antidiabetic effect of garcinol on streptozotocin-induced diabetic rats. Indian J. Pharm. Sci..

[35] Setiawan A., Hanum L., Wardoyo E.R.P. (2015). The effect of mundu fruit *Garcinia dulcis* Roxb Kurz methanol extract on lyphoprotein profile and trygliseride white rat *Rattus norvegicus* L.. J. Biol. Res..

[36] Liao C.H., Sang S., Liang Y.C., Ho C.T., Lin J.K. (2004). Suppression of inducible nitric oxide synthase and cyclooxygenase-2 in downregulating nuclear factor-kappa B pathway by garcinol. Mol. Carcinog..

[37] Madhuri K., Naik P.R. (2017). Modulatory effect of garcinol in streptozotocin-induced diabetic Wistar rats. Arch. Physiol. Biochem..

[38] Hung W.L., Tsai M.L., Sun P.P., Tsai C.Y., Yang C.C., Ho C.T., Cheng A.C., Pan M.H. (2014). Protective effects of garcinol on dimethylnitrosamine-induced liver fibrosis in rats. Food Funct..

[39] Bodhankar S.L., Kushawaha S.K., Thakurdesai P., Mohan V. (2016). Effect of cyclodextrin garcinol complex on isoproterenol-induced cardiotoxicity and cardiac hypertrophy in rats. Diabesity.

[40] Chan E.C., Dusting G.J., Guo N., Peshavariya H.M., Taylor C.J., Dilley R., Narumiya S., Jiang F. (2010). Prostacyclin receptor suppresses cardiac fibrosis: Role of CREB phosphorylation. J. Mol. Cell. Cardiol..

[41] Thongsepee N., Mahabusarakam W., Hiranyachattada S. (2017). Diuretic and hypotensive effect of morelloflavone from *Garcinia dulcis* in two-kidneys-one-clip (2K1C) hypertensive rat. Sains Malays..

[42] De Castro Moreira M.E., de Oliveira Araújo F., de Sousa A.R., Toledo R.C.L., dos Anjos Benjamin L., Veloso M.P., de Souza Reis K., dos Santos M.H., Martino H.S.D. (2018). Bacupari peel extracts (*Garcinia brasiliensis*) reduces the biometry, lipogenesis and hepatic steatosis in obese rats. Food Res. Int..

[43] Pinkaew D., Cho S.G., Hui D.Y., Wiktorowicz J.E., Hutadilok-Towatana N., Mahabusarakam W., Tonganunt M., Stafford L.J., Phongdara A., Liu M. (2009). Morelloflavone blocks injury-induced neointimal formation by inhibiting vascular smooth muscle cell migration. Biochim. Biophys. Acta.

[44] Lamai J., Mahabusarakam W., Ratithammatorn T., Hiranyachattada S. (2013). Effects of morelloflavone from *Garcinia dulcis* on vasorelaxation of isolated rat thoracic aorta. J. Physiol. Biomed. Sci..

[45] Verdam F.J., Fuentes S., de Jonge C., Zoetendal E.G., Erbil R., Greve J.W., Buurman W.A., de Vos W.M., Rensen S.S. (2013). Human intestinal microbiota composition is associated with local and systemic inflammation in obesity. Obesity.

[46] Lee P.S., Teng C.Y., Kalyanam N., Ho C.T., Pan M.H. (2019). Garcinol reduces obesity in high-fat-diet-fed mice by modulating gut microbiota composition. Mol. Nutr. Food Res..

[47] Goodman A.L., Kallstrom G., Faith J.J., Reyes A., Moore A., Dantas G., Gordon J.I. (2011). Extensive personal human gut microbiota culture collections characterized and manipulated in gnotobiotic mice. Proc. Natl. Acad. Sci. USA.

[48] An H.M., Park S.Y., Lee D.K., Kim J.R., Cha M.K., Lee S.W., Lim H.T., Kim K.J., Ha N.J. (2011). Antiobesity and lipid-lowering effects of *Bifidobacterium* spp. in high fat diet-induced obese rats. Lipids Health Dis..

[49] Le Chatelier E., Nielsen T., Qin J., Prifti E., Hildebrand F., Falony G., Almeida M., Arumugam M., Batto J.M., Kennedy S. (2013). Richness of human gut microbiome correlates with metabolic markers. Nature.

[50] Kameyama K., Itoh K. (2014). Intestinal colonization by a *Lachnospiraceae* bacterium contributes to the development of diabetes in obese mice. Microbes Environ..

[51] Song J.X., Ren H., Gao Y.F., Lee C.Y., Li S.F., Zhang F., Li L., Chen H. (2017). Dietary capsaicin improves glucose homeostasis and alters the gut microbiota in obese diabetic *ob*/*ob* mice. Front. Physiol..

[52] Jin M., Qian Z., Yin J., Xu W., Zhou X. (2019). The role of intestinal microbiota in cardiovascular disease. J. Cell. Mol. Med..

[53] Kasahara K., Tanoue T., Yamashita T., Yodoi K., Matsumoto T., Emoto T., Mizoguchi T., Hayashi T., Kitano N., Sasaki N. (2017). Commensal bacteria at the crossroad between cholesterol homeostasis and chronic inflammation in atherosclerosis. J. Lipid Res..

[54] Li Z., Wu Z., Yan J., Liu H., Liu Q., Deng Y., Ou C., Chen M. (2019). Gut microbe-derived metabolite trimethylamine N-oxide induces cardiac hypertrophy and fibrosis. Lab. Investig..

[55] Safari Z., Gérard P. (2019). The links between the gut microbiome and non-alcoholic fatty liver disease (NAFLD). Cell. Mol. Life Sci..

[56] Wong V.W., Tse C.-H., Lam T.T., Wong G.L., Chim A.M., Chu W.C., Yeung D.K., Law P.T., Kwan H.-S., Yu J. (2013). Molecular characterization of the fecal microbiota in patients with nonalcoholic steatohepatitis—A longitudinal study. PLoS ONE.

[57] Wang B., Jiang X., Cao M., Ge J., Bao Q., Tang L., Chen Y., Li L. (2016). Altered fecal microbiota correlates with liver biochemistry in nonobese patients with non-alcoholic fatty liver disease. Sci. Rep..

[58] Ma J., Zhou Q., Li H. (2017). Gut microbiota and nonalcoholic fatty liver disease: Insights on mechanisms and therapy. Nutrients.

[59] UNEP (2001). Citric Acid (CAS No: 77-92-9). United Nations Environment Programme.

[60] Majeed M., Bani S., Bhat B., Pandey A., Mundkur L., Neupane P. (2018). Safety profile of 40% garcinol from *Garcinia indica* in experimental rodents. Toxicol. Rep..

[61] Pang X., Yi T., Yi Z., Cho S.G., Qu W., Pinkaew D., Fujise K., Liu M. (2009). Morelloflavone, a biflavonoid, inhibits tumor angiogenesis by targeting rho GTPases and extracellular signal-regulated kinase signaling pathways. Cancer Res..

[62] Preuss H., Bagchi D., Bagchi M., Rao C., Dey D., Satyanarayana S. (2004). Effects of a natural extract of (–)-hydroxycitric acid (HCA-SX) and a combination of HCA-SX plus niacin-bound chromium and *Gymnema sylvestre* extract on weight loss. Diabetes Obes. Metab..

[63] Ohia S.E., Awe S.O., LeDay A.M., Opere C.A., Bagchi D. (2001). Effect of hydroxycitric acid on serotonin release from isolated rat brain cortex. Res. Commun. Mol. Pathol. Pharmacol..

[64] Ohia S.E., Opere C.A., LeDay A.M., Bagchi M., Bagchi D., Stohs S.J. (2002). Safety and mechanism of appetite suppression by a novel hydroxycitric acid extract (HCA-SX). Mol. Cell. Biochem..

[65] Walker E.B. (2007). HPLC analysis of selected xanthones in mangosteen fruit. J. Sep. Sci..

[66] Sekar S., Shafie S.R., Prasadam I., Crawford R., Panchal S.K., Brown L., Xiao Y. (2017). Saturated fatty acids induce development of both metabolic syndrome and osteoarthritis in rats. Sci. Rep..

[67] Parlee S.D., Lentz S.I., Mori H., MacDougald O.A., Macdougald O.A. (2014). Chapter Six—Quantifying size and number of adipocytes in adipose tissue. Methods in Enzymology.

[68] Schneider C.A., Rasband W.S., Eliceiri K.W. (2012). NIH Image to ImageJ: 25 years of image analysis. Nat. Methods.

[69] Good C.A., Kramer H., Somogyi M. (1933). The determination of glycogen. J. Biol. Chem..

[70] Goth L. (1991). A simple method for determination of serum catalase activity and revision of reference range. Clin. Chim. Acta.

[71] Folch J., Lees M., Sloane Stanley G. (1957). A simple method for the isolation and purification of total lipides from animal tissues. J. Biol. Chem..

[72] Zhou J., Bruns M.A., Tiedje J.M. (1996). DNA recovery from soils of diverse composition. Appl. Environ. Microbiol..

[73] Illumina (2013). MiSeq System User Guide.

[74] Bolger A.M., Lohse M., Usadel B. (2014). Trimmomatic: A flexible trimmer for Illumina sequence data. Bioinformatics.

[75] Edgar R.C. (2010). Search and clustering orders of magnitude faster than BLAST. Bioinformatics.

[76] Wemheuer B., Wemheuer F., Streit W.R., Daniel R. (2017). Assessing Bacterial and Fungal Diversity in the Plant Endosphere. Metagenomics: Methods and Protocols.

[77] Edgar R.C. (2016). UNOISE2: Improved error-correction for Illumina 16S and ITS amplicon sequencing. bioRxiv.

[78] Edgar R.C., Haas B.J., Clemente J.C., Quince C., Knight R. (2011). UCHIME improves sensitivity and speed of chimera detection. Bioinformatics.

[79] Quast C., Pruesse E., Yilmaz P., Gerken J., Schweer T., Yarza P., Peplies J., Glöckner F.O. (2013). The SILVA ribosomal RNA gene database project: Improved data processing and web-based tools. Nucleic Acids Res..

[80] Camacho C., Coulouris G., Avagyan V., Ma N., Papadopoulos J., Bealer K., Madden T.L. (2009). BLAST+: Architecture and applications. BMC Bioinform..

[81] Oksanen J., Blanchet F.G., Kindt R., Legendre P., Minchin P.R., O’hara R.B., Simpson G.L., Solymos P., Stevens M.H., Wagner H. (2018). Vegan: Community Ecology Package; R Package Version 2.5-2. https://CRAN.R-project.org/package=vegan.

[82] Oksanen J., Blanchet F.G., Friendly M., Kindt R., Legendre P., McGlinn D., Minchin P.R., O’Hara R.B., Simpson G.L., Solymos P. (2017). Vegan: Community Ecology Package; R Package Version 2.4-2. https://cran.r-project.org/.

[83] Clarke K.R., Gorley R.N. (2006). PRIMER v6: User Manual/Tutorial.

[84] Anderson M.J. (2001). A new method for non-parametric multivariate analysis of variance. Austral Ecol..

[85] Wang Y., Naumann U., Wright S.T., Warton D.I. (2012). mvabund—An R package for model-based analysis of multivariate abundance data. Methods Ecol. Evol..

[86] Asyifah M.R., Lu K., Ting H.L., Zhang D. (2014). Hidden potential of tropical fruit waste components as a useful source of remedy for obesity. J. Agric. Food Chem..

