# Supplementary material for: Physiological and Metabolic Effects of Yellow Mangosteen (Garcinia dulcis) Rind in Rats with Diet-Induced Metabolic Syndrome
Source: Int J Mol Sci. 2019 Dec 31;21(1):272. doi: 10.3390/ijms21010272 (PMC6981489; doi:10.3390/ijms21010272)
Supplement: Supplementary file 1 [file ijms-21-00272-s001.pdf]

International Journal of *Molecular Sciences*

MDPI

Article

Physiological and Metabolic Effects of Yellow Mangosteen (*Garcinia dulcis*) Rind in Rats With Diet-Induced Metabolic Syndrome

Oliver D. John [1,2], Peter Mouatt [3], Marwan E. Majzoub [4], Torsten Thomas [4], Sunil K. Panchal [1] and Lindsay Brown [1,2,*]

1 Functional Foods Research Group, University of Southern Queensland, Toowoomba, QLD 4350, Australia; oliverdjohn@outlook.com (O.D.J.), skpanchal2001@gmail.com (S.K.P.)
2 School of Health and Wellbeing, University of Southern Queensland, Toowoomba, QLD 4350, Australia
3 Southern Cross Plant Science, Southern Cross University, Lismore, NSW 2480, Australia; Peter.Mouatt@scu.edu.au (P.M.)
4 Centre for Marine Science and Innovation & School of Biological, Earth and Environmental Sciences, University of New South Wales, Sydney, NSW 2052, Australia; m.majzoub@unsw.edu.au (M.E.M), t.thomas@unsw.edu.au (T.T.)
* Correspondence: Lindsay.Brown@usq.edu.au (L.B.); Tel.: +61-7-4631-1319

Received: 14 November 2019; Accepted: 27 December 2019; Published: date

---

Supplementary File

International Journal of *Molecular Sciences*

MDPI

Supplementary Table S1. Relative abundance of zOTUs affected by diet (ANOVA with *p* adjusted <0.05) between C, CGD, H and HGD rats.

OTU_ID	C (%)	CGD (%)	H (%)	HGD (%)	Phylum	Family	Genus
Zotu24	0.89	1.43	0.04	0.02	Actinobacteria	Bifidobacteriaceae	*Bifidobacterium*
Zotu19	0.00	0.00	0.11	2.53	Firmicutes	Lachnospiraceae	[*Bacteroides*] pectinophilus group
Zotu228	0.17	0.15	0.00	0.00	Firmicutes	Lachnospiraceae	*Acetitomaculum*
Zotu131	0.05	0.05	0.36	0.25	Firmicutes	Lachnospiraceae	*Anaerostipes*
Zotu153	0.11	0.38	0.00	0.01	Firmicutes	Lachnospiraceae	*Lachnospiraceae* NK4A136 group
Zotu1122	0.00	0.00	0.02	0.02	Firmicutes	Lachnospiraceae	*Lachnospiraceae* NK4A136 group
Zotu118	0.00	0.01	0.81	0.11	Firmicutes	Lachnospiraceae	*Lachnospiraceae* UCG-006
Zotu714	0.00	0.00	0.04	0.03	Firmicutes	Lachnospiraceae	*Lachnospiraceae* UCG-008
Zotu162	0.00	0.01	0.26	0.23	Firmicutes	Lachnospiraceae	*Roseburia*
Zotu53	0.04	0.01	0.92	0.41	Firmicutes	Lachnospiraceae	unclassified
Zotu544	0.00	0.00	0.07	0.03	Firmicutes	Lachnospiraceae	unclassified
Zotu671	0.00	0.00	0.02	0.02	Firmicutes	Lachnospiraceae	unclassified
Zotu231	0.00	0.00	0.11	0.10	Firmicutes	Peptococcaceae	unclassified
Zotu70	0.02	0.06	0.62	0.15	Firmicutes	Peptostreptococcaceae	unclassified
Zotu166	0.12	0.17	0.00	0.00	Firmicutes	Ruminococcaceae	[*Eubacterium*] coprostanoligenes group
Zotu194	0.00	0.00	0.19	0.07	Firmicutes	Ruminococcaceae	*Ruminiclostridium 9*
Zotu272	0.01	0.01	0.05	0.09	Firmicutes	Ruminococcaceae	*Ruminiclostridium 9*
Zotu276	0.01	0.00	0.13	0.06	Firmicutes	Ruminococcaceae	*Ruminiclostridium 9*
Zotu236	0.00	0.00	0.18	0.06	Firmicutes	Ruminococcaceae	unclassified

Differential abundance analysis was performed using Mvabund. C, corn starch diet-fed rats; CGD, corn starch diet-fed rats treated with *Garcinia dulcis* rind powder; H, high-carbohydrate, high-fat diet-fed rats; HGD, high-carbohydrate, high-fat diet-fed rats treated with *Garcinia dulcis* rind powder.

Supplementary Table S2. Relative abundance of zOTUs affected by treatment (ANOVA with *p* adjusted <0.05) between C, CGD, H and HGD rats.

OTU_ID	C (%)	CGD (%)	H (%)	HGD (%)	Phylum	Family	Genus
Zotu28	0.09	0.78	0.06	0.75	Bacteroidetes	Tannerellaceae	*Parabacteroides*
Zotu46	0.08	0.86	0.02	0.36	Bacteroidetes	Tannerellaceae	*Parabacteroides*
Zotu211	0.01	0.17	0.01	0.07	Bacteroidetes	Tannerellaceae	*Parabacteroides*
Zotu343	0.00	0.07	0.00	0.03	Bacteroidetes	Tannerellaceae	*Parabacteroides*
Zotu477	0.00	0.06	0.00	0.01	Bacteroidetes	Tannerellaceae	*Parabacteroides*
Zotu604	0.00	0.01	0.00	0.02	Bacteroidetes	Tannerellaceae	*Parabacteroides*
Zotu3	9.24	0.07	13.31	1.19	Firmicutes	Clostridiaceae 1	*Clostridium sensu stricto 1*
Zotu1064	0.54	0.10	0.89	0.11	Firmicutes	Clostridiaceae 1	*Clostridium sensu stricto 1*
Zotu410	0.03	0.00	0.11	0.00	Firmicutes	Lachnospiraceae	*Acetitomaculum*
Zotu372	0.01	0.00	0.12	0.01	Firmicutes	Lachnospiraceae	*Blautia*
Zotu156	0.00	0.00	0.72	0.00	Firmicutes	Lachnospiraceae	*Lachnospiraceae* NK4A136 group
Zotu232	0.29	0.00	0.06	0.00	Firmicutes	Lachnospiraceae	*Lachnospiraceae* NK4A136 group
Zotu239	0.09	0.00	0.29	0.00	Firmicutes	Lachnospiraceae	*Lachnospiraceae* NK4A136 group
Zotu400	0.01	0.00	0.19	0.00	Firmicutes	Lachnospiraceae	*Lachnospiraceae* NK4A136 group
Zotu447	0.09	0.00	0.12	0.00	Firmicutes	Lachnospiraceae	unclassified
Zotu469	0.00	0.05	0.01	0.04	Firmicutes	Lachnospiraceae	unclassified
Zotu473	0.00	0.03	0.00	0.06	Firmicutes	Lachnospiraceae	unclassified
Zotu487	0.00	0.03	0.00	0.05	Firmicutes	Lachnospiraceae	unclassified
Zotu658	0.00	0.02	0.00	0.03	Firmicutes	Lachnospiraceae	unclassified
Zotu124	0.13	0.00	0.38	0.00	Firmicutes	Lachnospiraceae	unclassified
Zotu173	0.03	0.00	0.47	0.00	Firmicutes	Lachnospiraceae	unclassified
Zotu174	0.03	0.00	0.43	0.01	Firmicutes	Lachnospiraceae	unclassified
Zotu184	0.00	0.21	0.00	0.11	Firmicutes	Lachnospiraceae	unclassified
Zotu304	0.01	0.08	0.02	0.10	Firmicutes	Lachnospiraceae	unclassified
Zotu415	0.03	0.01	0.11	0.00	Firmicutes	Lachnospiraceae	unclassified
Zotu555	0.02	0.00	0.04	0.00	Firmicutes	Ruminococcaceae	*Pygmaiobacter*
Zotu392	0.03	0.00	0.06	0.02	Firmicutes	Ruminococcaceae	*Ruminococcaceae* UCG-010
Zotu155	0.36	0.00	0.03	0.00	Firmicutes	Ruminococcaceae	*Ruminococcaceae* UCG-014

Zotu328	0.13	0.00	0.03	0.00	Firmicutes	Ruminococcaceae	*Ruminococcaceae* UCG-014
Zotu1018	0.01	0.00	0.04	0.00	Firmicutes	Ruminococcaceae	*Ruminococcaceae* UCG-014
Zotu662	0.05	0.00	0.03	0.00	Firmicutes	Ruminococcaceae	UBA1819
Zotu412	0.02	0.00	0.10	0.00	Firmicutes	Ruminococcaceae	unclassified
Zotu249	0.07	0.00	0.13	0.00	Patescibacteria	Saccharimonadaceae	*Candidatus Saccharimonas*
Zotu21	0.12	1.66	0.01	0.34	Proteobacteria	Burkholderiaceae	*Parasutterella*
Zotu230	0.01	0.16	0.00	0.02	Proteobacteria	Burkholderiaceae	*Parasutterella*

Differential abundance analysis was performed using Mvabund. C, corn starch diet-fed rats; CGD, corn starch diet-fed rats treated with *Garcinia dulcis* rind powder; H, high-carbohydrate, high-fat diet-fed rats; HGD, high-carbohydrate, high-fat diet-fed rats treated with *Garcinia dulcis* rind powder.

International Journal of
Molecular Sciences

MDPI

Supplementary Table S3. PERMANOVAs based on Euclidean distance matrix for square-root transformed physiological data of all rat faecal samples.

Source	df	SS	MS	Pseudo-F	P(perm)	Unique perms
Diet	1	508800	508800	15.819	0.0002	9944
Treatment	1	1680200	1680200	52.238	0.0001	9929
Diet ×	1	80304	80304	2.4967	0.066	9941
Res	18	578960	578960			
Total	21	2829600				

PAIR-WISE TESTS

Groups	*t*	*p*(perm)	Unique perms
C, CGD	5.3183	0.0015	462
C, H	2.882	0.0083	126
C, HGD	3.0829	0.0022	462
CGD, H	7.593	0.0022	462
CGD, HGD	2.9817	0.0046	462
H, HGD	5.1842	0.0023	462

PERMDISP (PAIRWISE COMPARISONS)

Groups	*t*	*p*(perm)
C, CGD	2.4667	0.034
C, H	1.2354	0.2938
C, HGD	0.4574	0.6399
CGD, H	3.7652	0.0028
CGD, HGD	1.6279	0.2023
H, HGD	1.6442	0.151

p values were calculated using 9,999 permutations under a residual model. C, corn starch diet-fed rats; CGD, corn starch diet-fed rats treated with *Garcinia dulcis* rind powder; H, high-carbohydrate, high-fat diet-fed rats; HGD, high-carbohydrate, high-fat diet-fed rats treated with *Garcinia dulcis* rind powder.

Supplementary Table S4. Correlation between bacterial community structure and physiological parameters

Physiological Variables	R^2	*p* value
Feed efficiency	0.78	0.001
Body weight	0.75	0.001
Left ventricle and septum weight	0.66	0.002
Omental fat	0.64	0.001
Water intake	0.64	0.002
Total abdominal fat	0.59	0.001
Plasma non-esterified fatty acids	0.58	0.001
Retroperitoneal fat	0.57	0.001
Liver wet weight	0.57	0.004
Plasma triglycerides	0.57	0.002
Fat mass	0.57	0.001
Kidneys wet weight	0.52	0.004
Oral glucose tolerance area under the curve	0.40	0.012
Epididymal fat	0.38	0.015
Energy intake	0.36	0.020
Systolic blood pressure	0.35	0.026
Food intake	0.32	0.033

International Journal of *Molecular Sciences*

MDPI

Supplementary Table 5. Taxonomic assignments of the OTUs strongly correlated with physiological parameters.

OTU_ID	Phylum	Family	Genus	Correlation with physiological parameters
Zotu24	Actinobacteria	Bifidobacteriaceae	*Bifidobacterium*	Body weight (+), epididymal fat (+), fat mass (+), feed efficiency (+), Plasma non-esterified fatty
Zotu477	Bacteroidetes	Tannerellaceae	*Parabacteroides*	Kidney wet weight (+),Plasma non-esterified fatty acids (+), oral glucose tolerance area under the
Zotu343	Bacteroidetes	Tannerellaceae	*Parabacteroides*	Kidney wet weight (+), liver wet weight (+)
Zotu46	Bacteroidetes	Tannerellaceae	*Parabacteroides*	Kidney wet weight (+), liver wet weight (+)
Zotu211	Bacteroidetes	Tannerellaceae	*Parabacteroides*	Kidney wet weight (+)
Zotu28	Bacteroidetes	Tannerellaceae	*Parabacteroides*	Liver wet weight (+)
Zotu1064	Firmicutes	Clostridiaceae 1	*Clostridium sensu stricto 1*	Kidney wet weight (-), liver wet weight (-),(+), oral glucose tolerance area under the curve (-),
Zotu3	Firmicutes	Clostridiaceae 1	*Clostridium sensu stricto 1*	Liver wet weight (-), oral glucose tolerance area under the curve (-)
Zotu410	Firmicutes	Lachnospiraceae	*Acetitomaculum*	Kidney wet weight (-), liver wet weight (-), plasma triglycerides (-)
Zotu228	Firmicutes	Lachnospiraceae	*Acetitomaculum*	Left ventricle and septum wet weight (+), omental fat (+)
Zotu131	Firmicutes	Lachnospiraceae	*Anaerostipes*	Body weight (-), epididymal fat (-), fat mass (-), feed efficiency (-), left ventricle and septum wet
Zotu372	Firmicutes	Lachnospiraceae	*Blautia*	Kidney wet weight (-)
Zotu247	Firmicutes	Lachnospiraceae	*Coprococcus 2*	Energy intake (-), kidney wet weight (-)
Zotu1122	Firmicutes	Lachnospiraceae	*Lachnospiraceae* NK4A136 group	Body weight (-), epididymal fat (-), fat mass (-), feed efficiency (-), left ventricle and septum wet
Zotu153	Firmicutes	Lachnospiraceae	*Lachnospiraceae* NK4A136 group	Plasma non-esterified fatty acids (+), oral glucose tolerance area under the curve (+), omental fat (+),
Zotu400	Firmicutes	Lachnospiraceae	*Lachnospiraceae* NK4A136 group	Energy intake (-), food intake (-), kidney wet weight (-)
Zotu156	Firmicutes	Lachnospiraceae	*Lachnospiraceae* NK4A136 group	Food intake (-), kidney wet weight (-)
Zotu118	Firmicutes	Lachnospiraceae	*Lachnospiraceae* UCG-006	Energy intake (-), fat mass (-), feed efficiency (-)
Zotu714	Firmicutes	Lachnospiraceae	*Lachnospiraceae* UCG-008	Body weight (-), fat mass (-), feed efficiency(-), left ventricle & septum wet weight (-)
Zotu162	Firmicutes	Lachnospiraceae	*Roseburia*	Body weight (-), fat mass (-), feed efficiency (-), plasma non-esterified fatty acids (-), omental fat (-),
Zotu544	Firmicutes	Lachnospiraceae	unclassified	Body weight (-), energy intake (-), fat mass (-), food intake (-), feed efficiency (-), left ventricle and
Zotu184	Firmicutes	Lachnospiraceae	unclassified	Food intake (+), kidney wet weight (+), liver wet weight (+),plasma non-esterified fatty acids (+),oral
Zotu671	Firmicutes	Lachnospiraceae	unclassified	Body weight (-), fat mass (-), feed efficiency (-), left ventricle and septum wet weight (-), water
Zotu53	Firmicutes	Lachnospiraceae	unclassified	Body weight (-), energy intake (-), fat mass (-), feed efficiency (-)
Zotu415	Firmicutes	Lachnospiraceae	unclassified	Kidney wet weight (-), liver wet weight (-),systolic blood pressure (-)
Zotu304	Firmicutes	Lachnospiraceae	unclassified	Liver wet weight (+), oral glucose tolerance area under the curve (+)
Zotu469	Firmicutes	Lachnospiraceae	unclassified	Liver wet weight (+), oral glucose tolerance area under the curve (+)
Zotu173	Firmicutes	Lachnospiraceae	unclassified	Kidney wet weight (-)

Zotu174	Firmicutes	Lachnospiraceae	unclassified	Kidney wet weight (-)
Zotu473	Firmicutes	Lachnospiraceae	unclassified	Liver wet weight (+)
Zotu658	Firmicutes	Lachnospiraceae	unclassified	Liver wet weight (+)
Zotu70	Firmicutes	Peptostreptococcaceae	unclassified	Energy intake (-), fat mass (-), feed efficiency (-), food efficiency (-), omental fat (-)
Zotu166	Firmicutes	Ruminococcaceae	[*Eubacterium*] coprostanoligenes group	Body weight (+), fat mass (+)
Zotu555	Firmicutes	Ruminococcaceae	*Pygmaiobacter*	Kidney wet weight (-), plasma triglycerides (-)
Zotu276	Firmicutes	Ruminococcaceae	*Ruminiclostridium 9*	Body weight (-), fat mass (-), omental fat (-), total abdominal fat (-)
Zotu392	Firmicutes	Ruminococcaceae	*Ruminococcaceae* UCG-010	Kidney wet weight (-), plasma triglycerides (-)
Zotu1018	Firmicutes	Ruminococcaceae	*Ruminococcaceae* UCG-014	Energy intake (-), fat mass (-), food intake (-)
Zotu412	Firmicutes	Ruminococcaceae	unclassified	Kidney wet weight (-)
Zotu21	Proteobacteria	Burkholderiaceae	*Parasutterella*	Plasma non-esterified fatty acids (+), plasma triglycerides (+)
Zotu230	Proteobacteria	Burkholderiaceae	*Parasutterella*	Plasma non-esterified fatty acids (+), plasma triglycerides (+)

This table includes the physiological parameters strongly correlated ($p<0.05$) with the bacterial community and incorporates OTUs that interact with at least 3 of these parameters. Plus sign (+) indicates positive correlations, while minus sign (-) indicates negative correlations.

International Journal of *Molecular Sciences*

Supplementary Figure S1

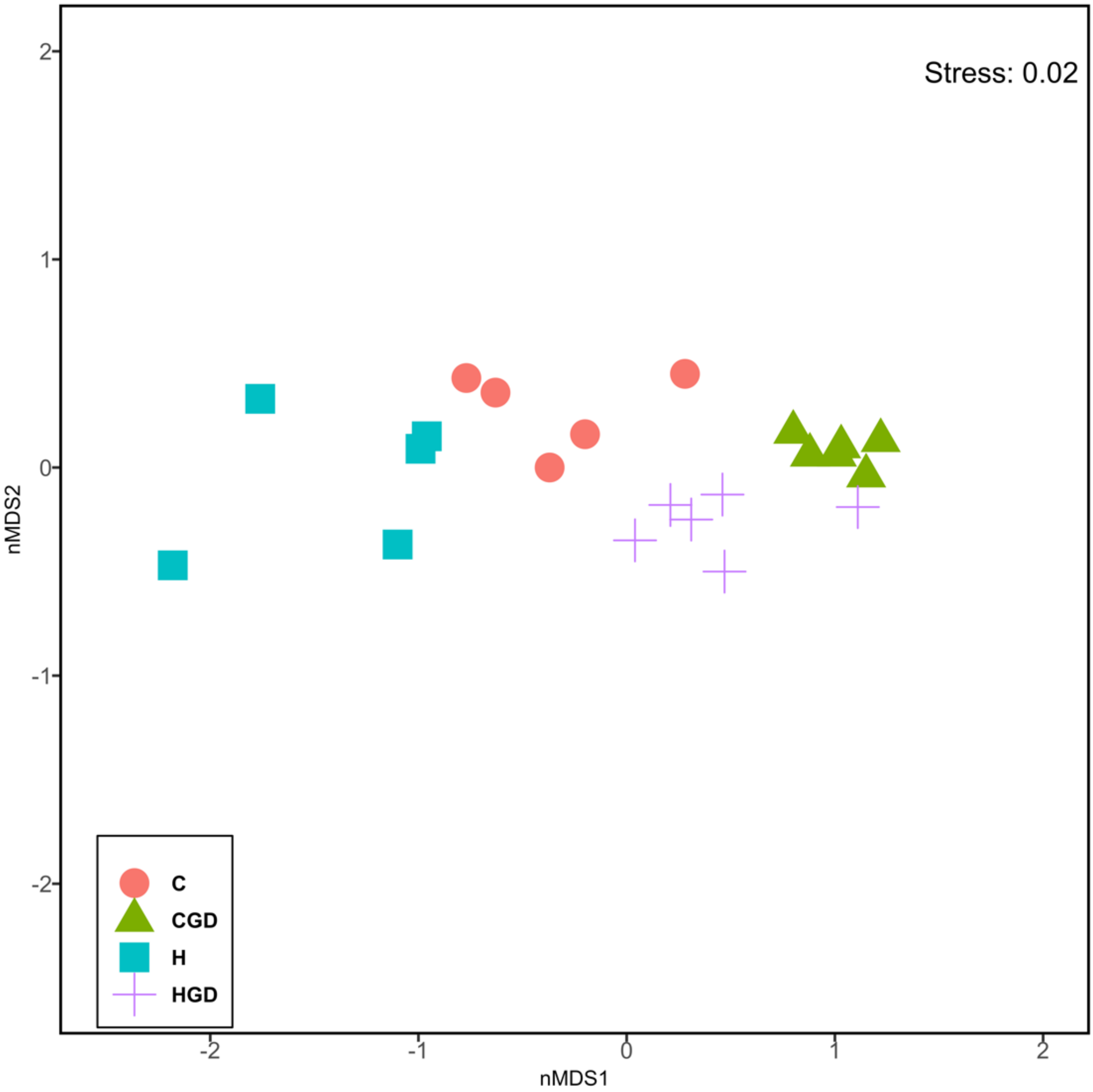

Supplementary Figure S1. nMDS plot of physiological data from 23 physiological parameters. C, corn starch diet-fed rats; CGD, corn starch diet-fed rats treated with *Garcinia dulcis* rind powder; H, high-carbohydrate, high-fat diet-fed rats; HGD, high-carbohydrate, high-fat diet-fed rats treated with *Garcinia dulcis* rind powder.

Supplementary Figure S2

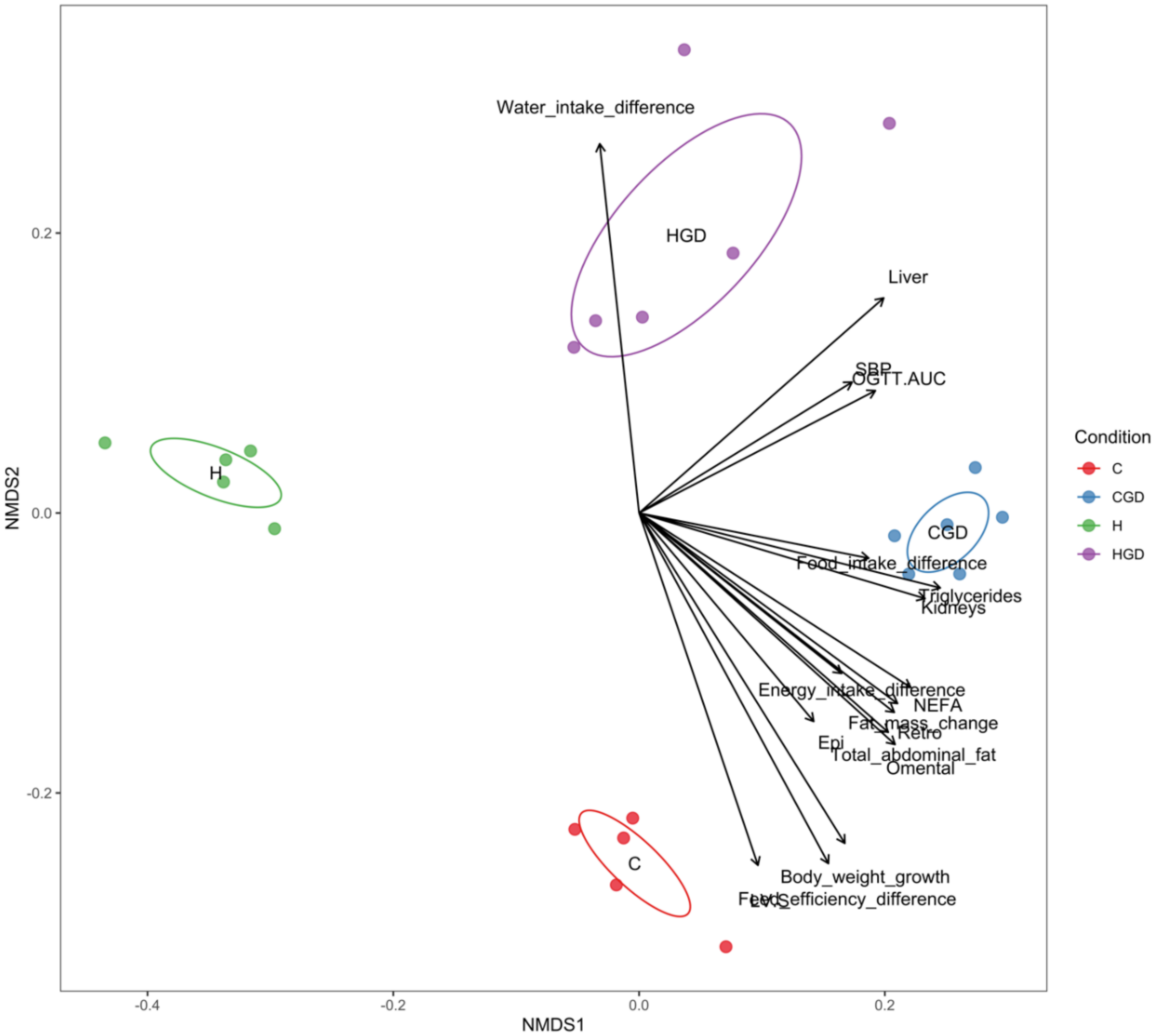

Supplementary Figure S2. Correlation between bacterial community structure (points) and environmental variables (arrows). C, corn starch diet-fed rats; CGD, corn starch diet-fed rats treated with *Garcinia dulcis* rind powder; H, high-carbohydrate, high-fat diet-fed rats; HGD, high-carbohydrate, high-fat diet-fed rats treated with *Garcinia dulcis* rind powder.

© 2019 by the authors. Submitted for possible open access publication under the terms and conditions of the Creative Commons Attribution (CC BY) license (http://creativecommons.org/licenses/by/4.0/).
